# Gene body methylation regulates gene expression and mediates phenotypic diversity in natural *Arabidopsis* populations

**DOI:** 10.1038/s41477-025-02108-4

**Published:** 2025-09-12

**Authors:** Zaigham Shahzad, Elizabeth Hollwey, Jonathan D. Moore, Jaemyung Choi, Gaëlle Cassin-Ross, Hatem Rouached, Matthew R. Robinson, Daniel Zilberman

**Affiliations:** 1 Department of Cell and Developmental Biology, John Innes Centre, Norwich, UK; 2 Department of Life Sciences, Syed Babar Ali School of Science and Engineering, Lahore University of Management Sciences, Lahore, Pakistan; 3 Institute of Science and Technology, Klosterneuburg, Austria; 4 Plant Resilience Institute, Michigan State University, East Lansing, MI USA; 5 Department of Plant, Soil, and Microbial Sciences, Michigan State University, East Lansing, MI USA

**Keywords:** Natural variation in plants, DNA methylation, Genetic variation

## Abstract

Genetic variation is generally regarded as a prerequisite for evolution. In principle, epigenetic information inherited independently of DNA sequence can also enable evolution, but whether this occurs in natural populations is unknown. Here we show that single-nucleotide and epigenetic gene body DNA methylation (gbM) polymorphisms explain comparable amounts of expression variance in *Arabidopsis thaliana* populations. We genetically demonstrate that gbM regulates transcription, and we identify and genetically validate many associations between gbM polymorphism and the variation of complex traits: fitness under heat and drought, flowering time and accumulation of diverse minerals. Epigenome-wide association studies pinpoint trait-relevant genes with greater precision than genetic association analyses, probably due to reduced linkage disequilibrium between gbM variants. Finally, we identify numerous associations between gbM epialleles and diverse environmental conditions in native habitats, suggesting that gbM facilitates adaptation. Overall, our results indicate that epigenetic methylation variation fundamentally shapes phenotypic diversity in a natural population.

## Abstract (web summary)

This study shows that epigenetic gene body DNA methylation regulates transcription and accounts for much of its variance in the *Arabidopsis* population, efficiently identifies genes regulating diverse traits and may facilitate local adaptation.

## Main

The neo-Darwinian or modern synthesis at the centre of evolutionary biology^1^ posits that DNA sequence changes are the substrate for evolution, with mechanisms such as natural selection and genetic drift shaping this variation to influence adaptation^2,3^. Epigenetic information, which can be encoded independently of the DNA sequence, is essential for cell fate determination, development and environmental responses in eukaryotes^4–7^. In theory, stably heritable epigenetic variation could contribute to adaptation^8–12^. Epiallelic variation in many angiosperm genes, including *Linaria vulgaris Cyc*, tomato *CNR* and *VTE3*, maize *Spm*, rice *D1*, oil palm *MANTLED* and *Arabidopsis thaliana FWA*, *PAI2* and *IAA7*, influences traits^13,14^. However, such epialleles are generally either too unstable to influence a response to selection^10–12^ (such as *Cyc*^15^, *D1*^16^ and *MANTLED*^17^), have an underlying genetic basis (such as *PAI2*^18^ and *IAA7*^14^) or are artificial (such as *FWA*^19^ and *MANTLED*^17^) or evidence is lacking that heritable epiallelic variation occurs in nature (such as *CNR*^20^, *VTE3*^21^, *Spm*^22^ and *D1*^16^). Furthermore, disentangling the effects of genetic and potentially epigenetic polymorphism in plant populations has proven difficult^23,24^, with most polymorphism that might be epigenetic instead attributed to local (*cis*) or distant (*trans*) genetic polymorphism^25^. Thus, the extent to which epigenetic inheritance mediates phenotypic diversity or influences evolutionary outcomes within natural populations is presently unclear^13,25,26^.

DNA methylation can be epigenetically inherited over many generations^13,27^ and occurs in transposable elements (TEs) and bodies of transcribed genes^28–31^. Plant TEs are methylated in all sequence contexts—CG, CHG and CHH (H being A, T or C)^7,28,29,32^. TE methylation induces silencing^32^, confers genome stability^31,33^ and can influence the expression of neighbouring genes^14,34–38^, and its variation has been associated with all known epialleles^13,26^. Gene body methylation (gbM) occurs only in the CG context^28,29,39^, although genes can also feature TE-like methylation in all contexts (teM)^40,41^. TeM is associated with silencing^30,40,41^, but the function of gbM has been extensively debated^42^. GbM is nearly ubiquitous in flowering plants^43,44^ and is common in animals^28,29,45^. In both groups, gbM preferentially resides in nucleosome-wrapped DNA within the exons of conserved, constitutively transcribed genes^30,46–49^. Conservation and phenomenological coherence suggest important functions^45^. Indeed, gbM is associated with (small) gene expression differences within and between plant species^24,41,50–53^, represses aberrant intragenic transcripts^54^ and appears to be under natural selection^51,52,55^. Moreover, loss of methyltransferase function causes developmental abnormalities in honeybees^56^, animals in which methylation is principally restricted to gene bodies^57^. However, gbM alteration has not been causatively linked to changes in gene expression in plants or animals^30,58,59^, leading to the proposals that gbM is a non-functional and somewhat deleterious by-product of TE methylation (in plants)^30,59,60^ or has functions unrelated to gene expression (in animals)^58^. Thus, the functional and evolutionary importance of gbM has been mysterious and controversial.

The *Arabidopsis* population exhibits extensive variation in TE methylation, gbM and teM^40,41^. Methylation levels of natural accessions are associated with climate^40^, suggesting that methylation variation could contribute to adaptation. Furthermore, genetically induced methylation polymorphism can account for the inheritance of complex *Arabidopsis* traits^61–63^, and methylation changes have been linked to adaptation under artificial selection^64,65^. Variation in TE methylation and teM has been repeatedly linked to genetic variation^26^, but local gbM variation is primarily epigenetic^41,66^ and, hence, is a potential epigenetic mediator of phenotypic variation. However, natural methylation variation^24^, and gbM variation specifically^40^, were concluded to have limited contributions to gene expression variance in *Arabidopsis*. Thus, the extent to which variation of gbM or any other type of methylation underlies phenotypic diversity or drives the evolution of complex traits in natural populations is unknown^13^.

## Results

### GbM and teM are independent phenomena

**Fig. 1:**
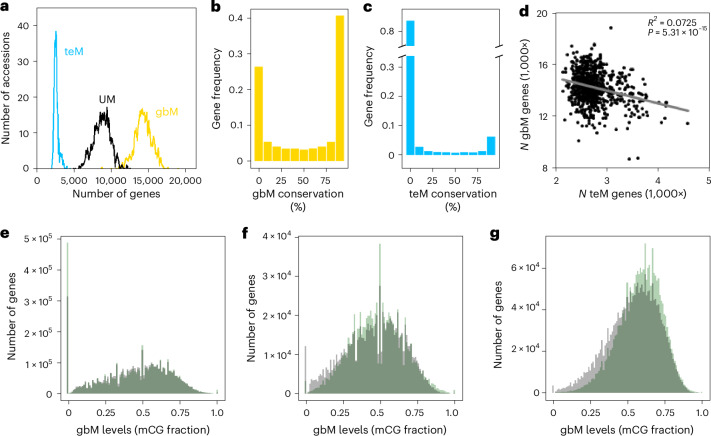
GbM and teM are independent phenomena. **a**, Frequency distributions of the number of genes classified as teM (blue), gbM (yellow) and UM (black) in 835 *Arabidopsis* accessions with >70% of genes called. **b**,**c**, Frequency distribution of gbM (**b**) and teM (**c**) conservation across 948 accessions within 24,465 genes with epigenetic state calls in >70% of accessions. **d**, Pearson’s correlation analysis between the number (*N*) of gbM and teM genes across accessions. **e**–**g**, Simulated (grey) and actual (green) mCG levels of all modelled genes (*N* = 6,736; **e**), genes with gbM frequency >99% and <100% in 740 accessions with global gbM similar to Col-0^66^ (*N* = 1,273; **f**) and genes with 100% gbM frequency (*N* = 2,942; **g**), across the 740 accessions or 740 simulation iterations, so that **e**, for example, shows the distribution of ~5 million (6,736 × 740) empirical and ~5 million simulated mCG data points.

**Extended Data Fig. 1:**
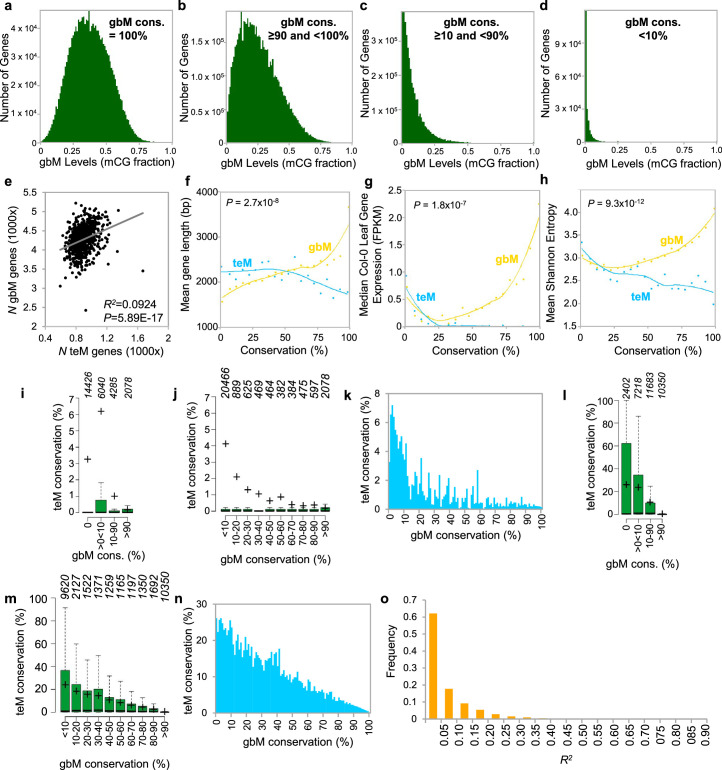
GbM and teM in *Arabidopsis.* **(a-d**) Distribution of gbM levels across 948 accessions for genes with 100% gbM population conservation (gbM in all accessions; N = 1884; **a**), gbM in ≥90 and <100% of accessions (N = 8119; **b**), gbM in ≥10 and <90% of accessions (N = 10,165; **c**), and gbM in <10% of accessions (N = 4973; **d**). Only genes with >20 CG sites are included. (**e**) Pearson’s correlation analysis between the number (*N*) of gbM and teM genes in accessions using published definitions of gbM and teM^60^. (**f**-**h**) Characteristics of genes binned by their gbM (gold) and teM (blue) frequencies: mean length in bp (**f**), median expression in Col-0 leaf RNA-seq data^54^ (**g**) and Shannon entropy^109^ (**h**). A linear model was used to associate gbM or teM population frequencies with the gene characteristics. *P*-values are for comparisons of gbM vs. teM associations using a two-sided F-test. (**i**-**k**) Conservation of teM epialleles in published^60^ gbM conservation categories (**i**, four gbM classes; **j**, decile gbM bins; **k**, percentile gbM bins). Analysis across three gbM conservation bins ( < 10%, 10-90%, and >90%) led to the published conclusion that teM frequency increases with gbM frequency^60^. However, we noted that the published <10% category contains only UM genes. Categorizing the published data^60^ in various ways (**i**-**k**) shows that teM prevalence decreases with increasing gbM. These results are broadly consistent with those obtained with our gbM and teM definitions: (**l**; four gbM classes), (**m**; decile gbM bins), and (**n**; percentile gbM bins). The numbers above box plots indicate the number of genes in each category, center lines represent sample medians, and plus signs correspond to means. Box limits indicate the 25th and 75th percentiles; whiskers extend to 1.5 times the interquartile range. Note that the numbers of genes in gbM conservation bins in panels **l** and **m** are different from those in Fig. 1b because only genes with epigenetic state calls in >70% of accessions are included in Fig. 1b, whereas this cutoff is not applied here. (**o**) Frequency distribution of correlation (*R*^2^) between gbM levels of individual genes and global gbM levels of accessions. A linear model is used to estimate *R*^2^.

Analyses of natural DNA methylation polymorphism in plant populations have not always strictly distinguished between gbM and teM, potentially motivated by the proposal that gbM is a by-product of teM^59^. To evaluate the relationship between gbM and teM, we categorized genes of 948 *Arabidopsis* accessions into three distinct epigenetic states: unmethylated (UM), gbM and teM using published data^40^ as previously described^54^. In brief, genes containing segments of only CG methylation (mCG) in a given accession were classed as gbM in that accession, those containing non-CG methylation segments were classed as teM and those containing neither and with sufficient sequence coverage were classed as UM^54^ (Supplementary Table 1 and Methods). Genes substantially overlapping both kinds of methylation segment (generally <1% of genes per accession) were classed as gbM and teM and excluded from further analyses. Considering unambiguously categorized genes, an accession contains on average 55% gbM genes, 33% UM genes and 12% teM genes (Fig. 1a). For example, the reference Col-0 accession has 56.5% gbM, 33.7% UM and 9.8% teM genes. Due to its variation, gbM is present in >90% of genes across the population (Supplementary Table 1). Consistent with published results^40,51^, we find that gbM conservation varies across genes, falling into three main groups: gbM in >90% of accessions (41% of genes), gbM in ≤90% and >10% of accessions (33%) and gbM in ≤10% of accessions (26%; Fig. 1b). Genes with high gbM population frequencies exhibit higher gbM levels that vary across a broader range (Extended Data Fig. 1a–d), as expected from the self-reinforcing gbM epigenetic dynamics^66^. In contrast to gbM, the vast majority of genes exhibit teM in ≤10% of accessions (Fig. 1c), suggesting that teM is disfavoured in most genes, probably due to its negative effects on expression^40^.

We find that the numbers of teM and gbM genes are very weakly (negatively) correlated across accessions (Fig. 1d) and are similarly weakly (positively) correlated under more restrictive definitions^60^ of gbM and teM (Extended Data Fig. 1e). Genes with higher gbM conservation tend to be long and are robustly and broadly transcribed^67,68^, the latter manifesting as high Shannon entropy (Extended Data Fig. 1f–h). By contrast, genes with higher teM conservation tend to be short and exhibit low expression and entropy (Extended Data Fig. 1f–h). TeM is most frequent in genes with low gbM conservation (Extended Data Fig. 1i–n). These results indicate that gbM and teM are prevalent in different types of genes and are not substantially associated. Consistently, a mathematical model that contains only gbM epigenetic dynamics accurately predicts gbM steady states and variation in *Arabidopsis*^66^. Using this model, we can precisely predict the distribution of gbM levels within a core set of 6,736 gbM genes across the *Arabidopsis* population, including the frequency at which genes are UM (Fig. 1e). The model can even make the subtle distinction between genes with 100% gbM population frequency and those that are gbM in >99% but <100% of accessions (Fig. 1f,g). In essence, we can computationally recapitulate the epigenetic evolution of *Arabidopsis* gbM without recourse to teM. These results do not support the hypotheses that gbM originates as a by-product of teM^59^ or that gbM promotes the transition to teM^60^. Instead, our data indicate that intragenic gbM and teM are largely independent and should be treated separately, which is consistent with many lineages having only TE methylation (fungi and some land plants) or only gbM (many invertebrates)^28–30^.

### GbM and teM explain substantial amounts of gene expression variance

A study attempting to partition expression variance attributable to genome-wide methylation variation versus single-nucleotide polymorphisms (SNPs) within 135 *Arabidopsis* accessions found that the effects of either methylation or SNPs could appear marginal^24^, presumably due to linkage disequilibrium between genetic and methylation polymorphisms^69^. A recent maize study also found it difficult to disentangle methylation and genetic variation^23^. To circumvent such limitations, we leveraged a statistical framework that robustly differentiates correlated variables^70^ to partition expression variance attributable to common SNPs, gbM and teM mCG polymorphisms within 625 *Arabidopsis* accessions for which methylation and expression data are available^40^.

**Fig. 2:**
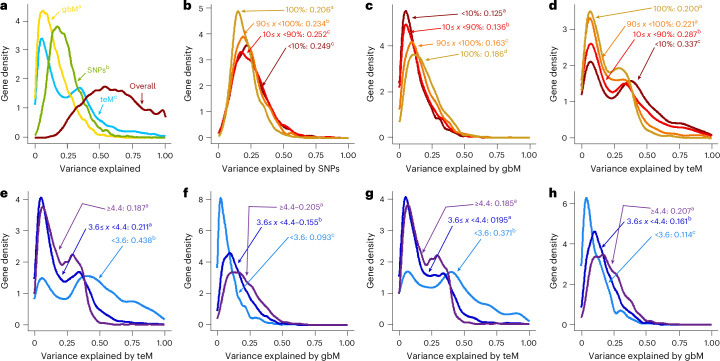
GbM and teM explain substantial amounts of gene expression variance. **a**, Density plots grouping successfully modelled genes (*N* = 7,339) by the proportion of the expression variation explained by genome-wide gbM (gold), genome-wide teM (blue), SNPs (green) or all three (brown). **b**–**d**, Genes were split by their population gbM frequency (<10%, *N* = 1,970; ≥10 and <90%, *N* = 1,508; ≥90 and <100%, *N* = 1,970; 100%, *N* = 1,891). The proportion of expression variation explained by SNPs (**b**), gbM (**c**) and teM (**d**) is plotted. **e**,**f**, Genes were split by Shannon entropy of expression (<3.6, *N* = 1,766; 3.6–4.4, *N* = 3,584; ≥4.4, *N* = 1,935), and the proportion of expression variation explained by teM (**e**) and gbM (**f**) is plotted. **g**,**h**, GbM genes (gbM population frequency ≥90%) were split by Shannon entropy (<3.6, *N* = 427; 3.6–4.4, *N* = 1,854; ≥4.4, *N* = 1,564) and the proportion of expression variation explained by teM (**g**) and gbM (**h**) is plotted. Superscript letters after mean values in all panels signify *P* < 0.01 using the non-parametric Kruskal–Wallis test followed by pairwise comparisons using the Wilcoxon rank-sum test with Bonferroni correction for multiple testing. Groups sharing the same letter are not significantly different.

We find that SNPs, gbM and teM explain substantial (and comparable) fractions of expression variance: SNPs explain 23.5% on average, gbM 15.2% and teM 26.0% (Fig. 2a). The variance attributable to SNPs is similar among genes with <90% gbM population frequency, with somewhat less variance explained in ≥90% gbM genes (Fig. 2b). By contrast, gbM explains considerably more expression variance as its population frequency increases (Fig. 2c). In genes with 100% gbM frequency, the effects of gbM (18.6%) and SNPs (20.6%) are nearly equal (Fig. 2b,c). TeM effects are bimodal (Fig. 2d), probably because they can be large but affect only a subset of genes due to teM rarity (Fig. 1a), so that teM expression effects are either substantial or effectively absent.

TeM explains more expression variance as gbM frequency decreases (Fig. 2d). Because we could only successfully model genes with low teM population frequencies (generally <3%; Supplementary Table 1), this effect is not due to differential *cis* teM prevalence. Instead, we find that teM explains more expression variance as Shannon entropy decreases (Fig. 2e), whereas gbM shows the opposite trend (Fig. 2f). We observe this even in genes with high gbM population frequencies (Fig. 2g,h), meaning that the trend is caused primarily by *trans* effects: gbM is more important for gene networks that regulate broadly and constitutively expressed genes, whereas teM is more important for networks regulating tissue-specific and inducible genes.

Although we find that teM and gbM explain substantial fractions of expression variance, the implications differ. Many *trans* genetic polymorphisms have been found to influence teM^40,41,50,71,72^, and teM variation has been repeatedly linked with local genetic variation^37,38,41^, especially structural variation (SV; insertions or deletions) caused by transposition. Hence, the extent to which teM variation is fundamentally epigenetic is unclear: much of it may be a readout for genetic variation. By contrast, although *trans* factors influence global gbM, local gbM variation is primarily caused by stochastic epigenetic fluctuations^66^. Consistently, gbM levels of individual genes are weakly associated with global gbM levels across accessions (*R*^2^ < 0.1 for ~80% genes; Extended Data Fig. 1o). Therefore, our gbM results indicate that much of the transcriptional variation in the *Arabidopsis* population is attributable to epigenetic inheritance.

### Local intragenic methylation polymorphism is associated with transcriptional variance

**Fig. 3:**
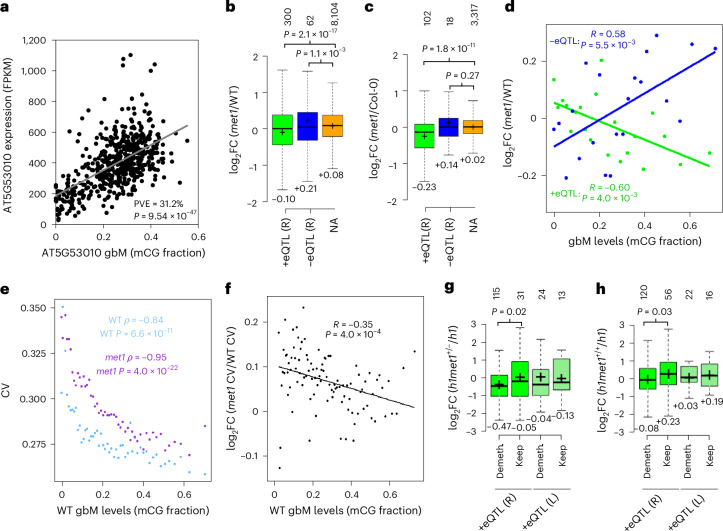
GbM quantitatively affects gene expression. **a**, GbM level and expression of *AT5G53010* across accessions. Per cent expression variance explained (PVE) by gbM is indicated. Pearson’s correlation analysis was used to assess the association between the two variables. FPKM, fragments per kilobase of transcript per million mapped reads. **b**,**c**, Expression in *met1* seedlings compared with WT of Bonferroni (*α* = 0.05) retained eQTL^gbM^ genes across 16 accessions (**b**) and across Col-0 tissues (leaf, seedling and inflorescence; **c**). Numbers of unique genes within each group are noted above the plots, means are indicated by ‘+’ and noted below the plots. Sample medians are shown by centre lines, and box edges represent the 25th and 75th percentiles. Whiskers extend to 1.5 times the interquartile range. *P* values were calculated using a two-tailed Student’s *t*-test to compare the indicated eQTL group with non-associated (NA) genes. **d**, Relationship between gbM levels of retained +eQTL^gbM^ and −eQTL^gbM^ genes in WT plants across 16 accessions and log_2_ fold expression change in *met1* compared with the WT of that accession. Genes were grouped by gbM levels. *R* and *P* values correspond to Pearson’s correlation. **e**, Relationship between the gene expression coefficient of variation (CV) across biological replicates in WT (blue) and *met1* (purple) and WT gbM level across 16 accessions. Genes were grouped by gbM levels in WT. *ρ* and *P* values correspond to Spearman’s rank correlation coefficient. **f**, Relationship between the log_2_ fold CV change in *met1* compared with WT and the gbM level across 16 accessions. *R* and *P* values correspond to Pearson’s correlation. **g**,**h**, Expression in *h1met1*^+*/−*^ (**g**) and *h1met1*^+*/+*^ (**h**) compared with *h1* of Bonferroni (*α* = 0.05) retained (R) or lost (L) eQTL^gbM^ genes that are either demethylated (Demeth.) or keep methylation. Box plots as in **b** and **c**. *P*, two-tailed Student’s *t*-test.

**Extended Data Fig. 2:**
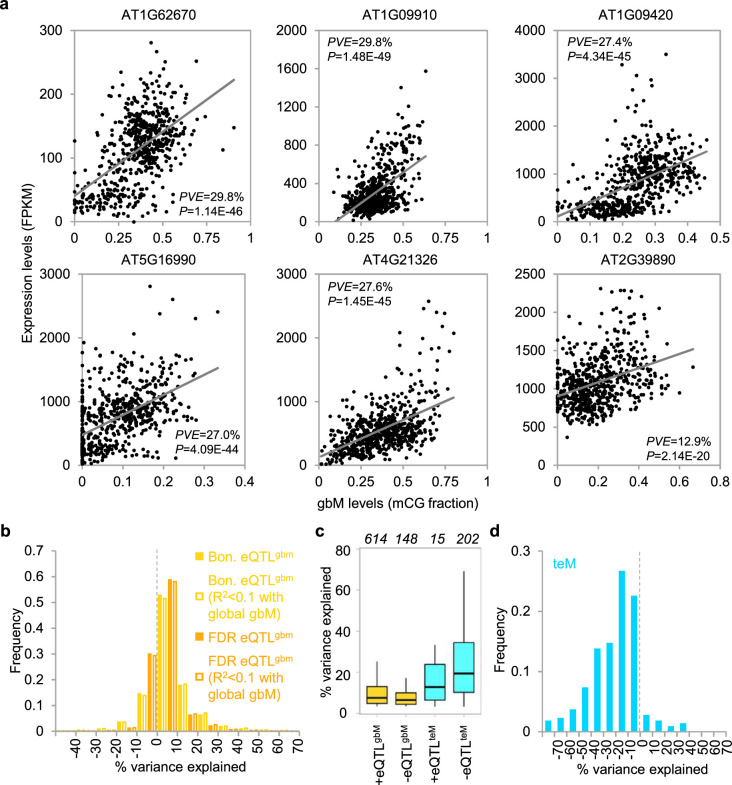
Association between gbM, teM and expression of *Arabidopsis* genes. (**a**) GbM and expression of six example genes across accessions. Percent expression variance explained (PVE) by gbM and *P* values of Pearson’s correlation tests are indicated. (**b**) Frequency distribution of percent expression variance of Bonferroni α = 0.05 and FDR 0.05 eQTL^gbM^ genes explained by mCG variation. The filled bars depict all eQTL^gbM^ genes significant at respective thresholds, and the empty bars represent eQTL^gbM^ genes showing *R*^2^ < 0.1 between local and global gbM levels. (**c**) Percent expression variance explained by gbM or teM in Bonferroni eQTL^gbM/teM^ genes. The number of QTLs corresponding to each category is indicated. Center lines represent sample medians, box limits indicate the 25th and 75th percentiles, whiskers extend to 1.5 times the interquartile range. (**d)** Frequency distribution of percent expression variance of Bonferroni eQTL^teM^ genes explained by mCG variation.

**Extended Data Fig. 3:**
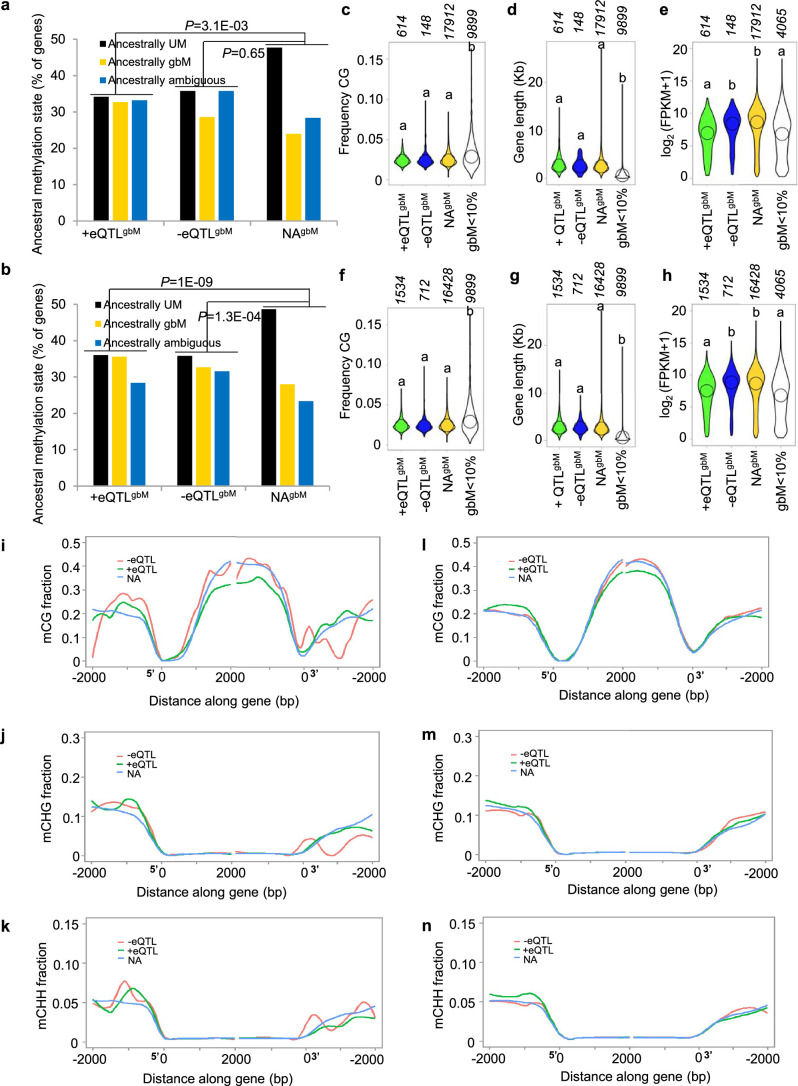
Characteristics of eQTL gbM genes. (**a**-**b**) Ancestral methylation states of +eQTL^gbM^, -eQTL^gbM^, and NA^gbM^ genes using the Bonferroni (**a**) or FDR 0.05 (**b**) classification. Ancestral methylation states were determined by analyzing methylation states of *Arabidopsis thaliana* orthologs in *Arabidopsis lyrata* and *Capsella rubella* and retrieved from^51^. *P* values correspond to chi-squared test. (**c**-**h**) Plots show CG dinucleotide frequency (**c** and **f**), gene length (**d** and **g**), and gene expression (**e** and **h**) of Bonferroni (**c-e**) or FDR 0.05 (**f-h**) eQTL^gbM^ genes, NA^gbM^ (non-associated) genes, and genes with gbM in <10% of accessions. Different letters signify *P* < 0.001, one-way ANOVA, Dunn’s test. Numbers of genes within each group are indicated. (**i**-**n**) Methylation in the CG (**i** and **l**), CHG (**j** and **m**) and CHH (**k** and **n**) contexts within and adjacent to +eQTL^gbM^, -eQTL^gbM^ and NA^gbM^ genes using the Bonferroni (**i-k**) or FDR 0.05 (**l-n**) classifications.

The above analyses (Fig. 2) indicate that gene expression variance is influenced by methylation in natural populations, but do not distinguish *cis* and *trans* effects. To identify functional *cis* gbM and teM epialleles, we analysed associations between mCG and mRNA levels of individual genes. We identified 614 +eQTL^gbM^ genes (eQTL stands for expression quantitative trait locus) that show a positive association between gbM and gene expression and 148 −eQTL^gbM^ genes that exhibit a negative association at a conservative significance threshold (Bonferroni *α* = 0.05); more eQTLs were identified at less stringent thresholds (Fig. 3a, Extended Data Fig. 2a and Supplementary Tables 2 and 3). The dominance of positive associations between local gbM and expression variation (Extended Data Fig. 2b,c) is consistent with findings from previous studies^24,41,50–53^. We find that eQTL^gbM^ genes are more likely to have had gbM before the speciation of *A. thaliana* than non-associated gbM (NA^gbM^) genes^51^ (Extended Data Fig. 3a,b), suggesting that they are under selection to retain gbM. CG dinucleotide composition and length—hallmark features of gbM genes^60^—are similar between eQTL^gbM^ and NA^gbM^ genes (Extended Data Fig. 3c–h), as are methylation patterns within and outside the genes (Extended Data Fig. 3i–n). However, gbM levels are slightly lower in +eQTL^gbM^ genes (Extended Data Fig. 3i,l), which also show lower expression (Extended Data Fig. 3e,h), suggesting that gbM may have more pronounced positive effects on gene expression when transcription is lower. In contrast to gbM, teM associations with expression are (as expected^40,41^) overwhelmingly negative (Extended Data Fig. 2c,d and Supplementary Table 2), consistent with teM and gbM exerting different effects on transcription.

**Extended Data Fig. 4:**
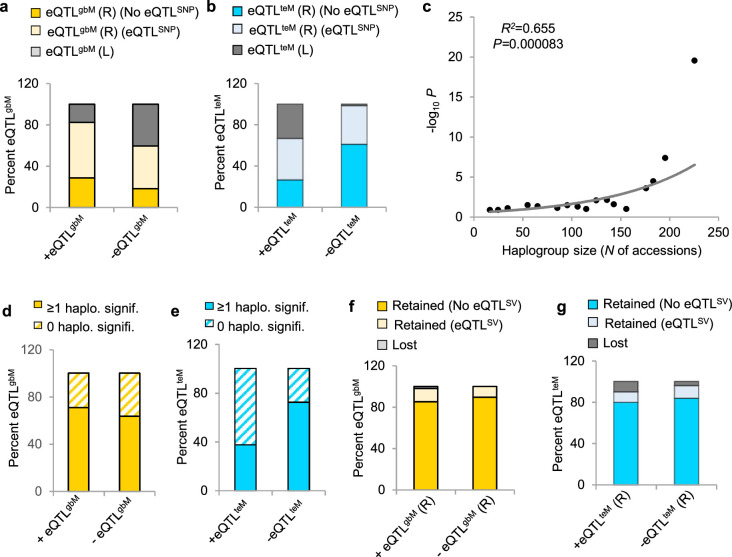
Most eQTL^gbM/teM^ gene expression effects are independent of local SNP variation. (**a** and **b**) Frequency of retained (R) or lost (L) eQTL^gbM^ (**a**) and eQTL^teM^ (**b**) genes after accounting for expression GWA SNPs. (**c**) Loss of statistical power for association analyses in haplogroups due to decreasing population size. The -log_10_ *P* values for associations between gbM/teM and gene expression exhibit an exponential increase with respect to the number of accessions in haplogroups. Indicated *R*^2^ and *P* values correspond to exponential regression. (**d** and **e**) Frequency of eQTL^gbM^ (**d**) and eQTL^teM^ (**e**) genes with a significant association between mCG and gene expression in at least one haplogroup. Despite reduced statistical power (**c**) in SNP invariant haplogroups due to smaller population sizes, significant associations between gbM variation and expression are detected for a majority of eQTL^gbM^ genes. (**f** and **g**) Frequency of retained or lost eQTL^gbM^ (**f**) and eQTL^teM^ (**g**) genes after accounting for structural variation (SV) effects on expression.

**Extended Data Fig. 5:**
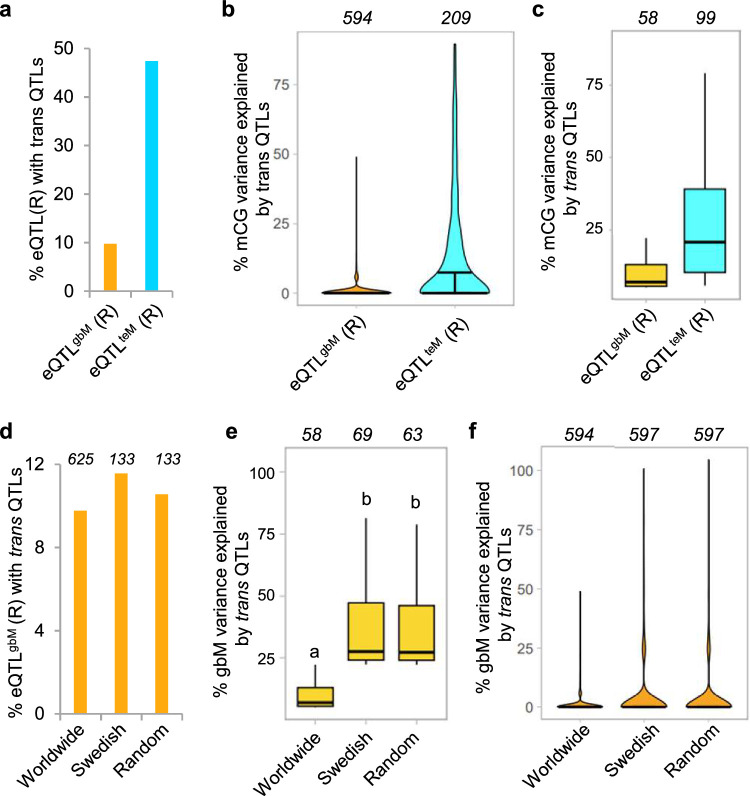
Trans genetic variation explains a minor fraction of gbM. (**a**) Percentage of eQTL^gbM/teM^ retained (R) genes that have *trans* genetic QTLs associated with the variation of gbM or teM. (**b**) Average effects ( ± standard error) of *trans* polymorphism on mCG variation in all retained eQTL^gbM/teM^ genes, with number of QTLs indicated. (**c**) Effect sizes of *trans* polymorphism on mCG variation of retained eQTL^gbM/teM^ genes having *trans* QTLs. Center lines represent sample medians, box limits indicate the 25th and 75th percentiles, whiskers extend to 1.5 times the interquartile range. The number of QTLs corresponding to each class is indicated above the plots. (**d**) Percentage of retained eQTL^gbM^ having *trans* QTLs in the entire worldwide population, a published Swedish population^50^, and a random population of equal size to the Swedish population. Numbers of accessions in each panel are indicated. (**e**) Effect sizes of *trans* genetic polymorphisms on gbM variation of retained eQTL^gbM^ genes having *trans* QTLs in worldwide, Swedish, and random populations. Box plots as in **c**. Different letters signify *P* < 0.05, one-way ANOVA, Tukey’s test. (**f**) Average effects ( ± standard error) of *trans* genetic variation on gbM variation of all retained eQTL^gbM^ genes in worldwide, Swedish, and random populations, with number of genes indicated. Note the inflation of estimated *trans* effects in the smaller populations, a phenomenon known as the Beavis effect^119,120^.

Given that genetic and epigenetic variation can be linked in the population^73^, we investigated whether methylation variants influence expression independently of *cis*-acting DNA sequence changes. We identified *cis* SNPs associated with expression of the eQTL^gbM/teM^ Bonferroni genes, and retained eQTL^gbM/teM^ if significant associations between methylation and expression variation persisted after accounting for *cis* SNPs associated with expression (Supplementary Fig. 1). Nearly all −eQTL^teM^ were retained, as were >80% of +eQTL^gbM^, and >60% of −eQTL^gbM^ and +eQTL^teM^ (Extended Data Fig. 4a,b and Supplementary Table 4). To account for residual confounding effects of SNPs, we defined SNP-invariant haplogroups for these genes and detected significant associations between mCG and gene expression for most eQTL^gbM^ and −eQTL^teM^ (Extended Data Fig. 4c–e and Supplementary Tables 5 and 6). Furthermore, we found the effects of known SV^74^ on eQTL^gbM^ to be negligible (Extended Data Fig. 4f), whereas eQTL^teM^ are more often lost after accounting for SV (Extended Data Fig. 4g), consistent with the known association between teM variation and TE SV^37,38,41^. In addition, we find that many (47.4%) retained eQTL^teM^ genes are affected by *trans* (presumably genetic) polymorphism (Extended Data Fig. 5a–c), which is consistent with published results^40,41,50,71,72^. By contrast, *trans* genetic variation accounts for only ~1% of gbM variance within eQTL^gbM^ (Extended Data Fig. 5d–f and Methods). These findings support the conclusion that epigenetic gbM variation explains substantial gene expression variance in the *Arabidopsis* population, whereas teM variation is often a readout for *cis* or *trans* genetic polymorphism. This distinction highlights the importance of analysing gbM variation for understanding expression diversity within plant populations.

### Loss of gbM quantitatively affects the expression of eQTL^gbM^ genes

**Extended Data Fig. 6:**
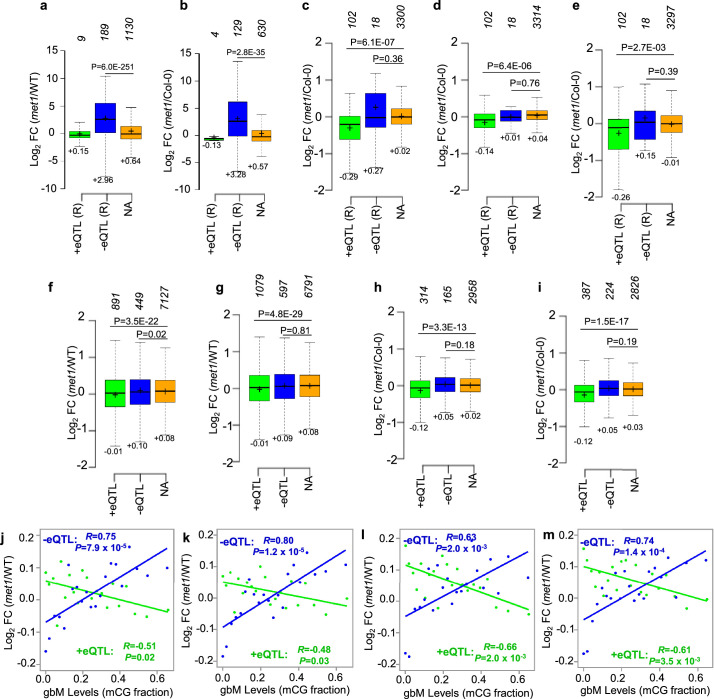
GbM quantitatively affects gene expression. (**a** and **b**) Expression in *met1* compared to WT of Bonferroni (α = 0.05) eQTL^teM^ genes retained (R) after accounting for genetic variation in seedlings across 16 accessions^75^ (**a**) and different tissues of Col-0 (leaf, seedling and inflorescence^54,77^; **b**). (**c**-**e**) Expression in *met1* compared to Col-0 of Bonferroni (α = 0.05) eQTL^gbM^ genes retained (R) after accounting for genetic variation in either leaf (**c**), inflorescence (**d**) or seedling (**e**). (**f** and **g**) Expression in *met1* compared to WT seedlings of FDR 0.01 (**f**) and FDR 0.05 (**g**) eQTL^gbM^ genes across 16 accessions. (**h** and **i**) Expression in *met1* compared to WT of FDR 0.01 (**h**) and FDR 0.05 (**i**) eQTL^gbM^ genes in different tissues of Col-0 (leaf, seedling and inflorescence). Center lines within box plots represent sample medians, plus signs correspond to means and are noted below the plots. Box limits indicate the 25th and 75th percentiles, whiskers extend to 1.5 times the interquartile range. Numbers of genes within each group are noted above the plots. *P*, two-tailed Student’s t-test between indicated group and NA (non-associated) genes. (**j**-**m**) Relationship between gbM levels of retained +eQTL and –eQTL genes using the FDR 0.01 (**j** and **l**) or FDR 0.05 (**k** and **m**) groups in WT plants across 16 accessions^75^ and log_2_ fold expression change in *met1* compared to WT of that accession. Genes with mCG >5% and/or non-CG methylation >1% in the putative promoter (2 kb upstream or up to the nearest upstream gene, whichever is shorter) were excluded in **l** and **m**. Genes were grouped by gbM levels. *R* and *P* values correspond to Pearson’s correlation.

To determine whether intragenic DNA methylation directly affects gene expression, we analysed published RNA sequencing (RNA-seq) data from *met1* mutants and wild-type (WT) controls across 16 natural *Arabidopsis* accessions^75^. Inactivation of the *MET1* methyltransferase causes complete loss of gbM and nearly complete loss of mCG throughout the genome^76^. WT methylated Bonferroni −eQTL^teM^ genes are strongly overexpressed in *met1* (Extended Data Fig. 6a), consistent with the established repressive activity of teM^30,40,41^. As expected from the associations, Bonferroni +eQTL^gbM^ genes are modestly downregulated (expressed at ~88% of WT compared with NA^gbM^ controls), whereas −eQTL^gbM^ genes are modestly upregulated (expressed at ~109% of WT compared with NA^gbM^ controls; Fig. 3b). Analysis of additional Col-0 *met1* seedling^54^, leaf^54^ and inflorescence^77^ RNA-seq datasets produced analogous results for −eQTL^teM^ and +eQTL^gbM^ genes, but −eQTL^gbM^ expression differences are not significant (probably due to the low number of these genes; Fig. 3c and Extended Data Fig. 6b–e). Analysis of genes that passed less stringent significance thresholds produced similar results, albeit with decreased effect sizes (Extended Data Fig. 6f–i). Furthermore, +eQTL^gbM^ genes with higher mCG show stronger downregulation in *met1* RNA-seq data, whereas −eQTL^gbM^ genes with higher mCG exhibit stronger upregulation (Fig. 3d and Extended Data Fig. 6j,k), indicating that gbM quantitatively affects gene expression. The quantitative relationship between WT gbM and *met1* expression remains after removal of genes with methylation in the putative promoter (Extended Data Fig. 6l,m). Although *MET1* inactivation could influence gene expression by altering non-CG methylation and histone modifications^78^, these chromatin features are not significantly changed in any relevant gbM gene category (Supplementary Fig. 2) and, thus, cannot explain our results.

The prevalence of gbM in constitutively expressed genes has motivated the proposal that gbM stabilizes gene expression by reducing transcriptional noise^45,67,68,79,80^, so that gbM effects on mRNA levels could be interpreted as a secondary consequence. To test this, we analysed interreplicate variance within the *met1* and WT RNA-seq data from 16 *Arabidopsis* accessions^75^. As expected, there is a strong negative correlation between transcriptional variability and gbM prevalence, but this remains the case in *met1* (Fig. 3e and Supplementary Fig. 3a). Variability is elevated in *met1*, but this effect is strongest in genes with low gbM, and decreases with gbM prevalence, including in eQTL^gbM^ genes (Fig. 3e,f and Supplementary Fig. 3). Given our observation that teM effects on expression also decrease with gbM prevalence (Fig. 2d), higher transcriptional variability in *met1* is probably caused by teM disruption. Therefore, any potential effects of gbM on transcriptional variability are low enough to be masked in *met1* data, whereas we can robustly detect gbM effects on steady-state mRNA levels in the same data (Fig. 3b,d and Extended Data Fig. 6f,g,j–m).

To further evaluate the direct impact of gbM loss on gene expression, we analysed a plant that is heterozygous for *met1* (*met1*^+/−^) and has relatively normal TE methylation and limited gbM loss^54^. This plant also contains loss-of-function mutations in two histone H1 genes^54^; therefore, expression was analysed with respect to *h1*-mutant controls. We analysed only +eQTL^gbM^ genes, as we lacked statistical power for the smaller number of −eQTL^gbM^ genes. Retained +eQTL^gbM^ genes demethylated in this plant have significantly decreased (~35%) expression compared with retained +eQTL^gbM^ genes that maintain gbM (Fig. 3g), specifically linking gbM loss with reduced expression. To validate these findings, we isolated six *h1met1*^+/+^ progeny of *h1met1*^+/−^. These plants exhibit mosaic demethylation of gbM genes, whereas TE methylation is comparatively normal (Supplementary Fig. 4). Retained +eQTL^gbM^ genes demethylated in *h1met1*^+/+^ plants display significantly reduced (~25%) expression compared with retained +eQTL^gbM^ genes that keep gbM (Fig. 3h). Altogether, we find that gbM loss consistently influences the expression of eQTL^gbM^ genes, regardless of the genetic background, tissue (seedlings, leaves or inflorescence), presence of functional *MET1*, or the extent of global teM or gbM perturbation. Therefore, our results establish gbM as a quantitative gene expression regulator.

### GbM variation enables efficient identification of new functional genes

We find that methylation polymorphism explains a substantial amount of natural expression variance and directly affects gene expression (Figs. 2 and 3). This implies that methylation epialleles should drive trait variation in natural populations. To uncover how DNA methylation shapes natural phenotypic diversity, we performed epigenome-wide association (epiGWA) analyses between gbM or teM polymorphism and the variation of complex traits: relative fitness under different conditions^81^, 9 flowering time-related traits^82^ and the accumulation of 18 minerals in leaves^83^. We identified 1 QTL^gbM^ for fitness in Madrid (hot climate) under low rainfall and high-density population growth (MLP), 8 QTL^gbM^ for flowering time traits and 19 QTL^gbM^ for leaf minerals (Supplementary Figs. 5–10, Supplementary Tables 7–13 and Methods). We also identified one QTL^teM^ for fitness in MLP conditions and six QTL^teM^ for mineral accumulation (Supplementary Figs. 5, 6 and 10 and Supplementary Tables 8 and 13). With the notable exception of two extensively studied flowering time genes—*FLC* and *FRI*^84^—there was virtually no overlap between QTL^gbM/teM^ and genetic QTLs (Supplementary Figs. 6, 9 and 10 and Supplementary Tables 13–15), suggesting distinct contributions of methylation variation to phenotypic diversity. Nonetheless, we found linkage disequilibrium^69^ (*r* = 0.725, *D*′ = 0.824, *P* < 0.0001) between *FRI* gbM and SNPs, suggesting that *FRI* epigenetic and genetic QTLs are redundant, and therefore we excluded *FRI* from further analyses.

**Fig. 4:**
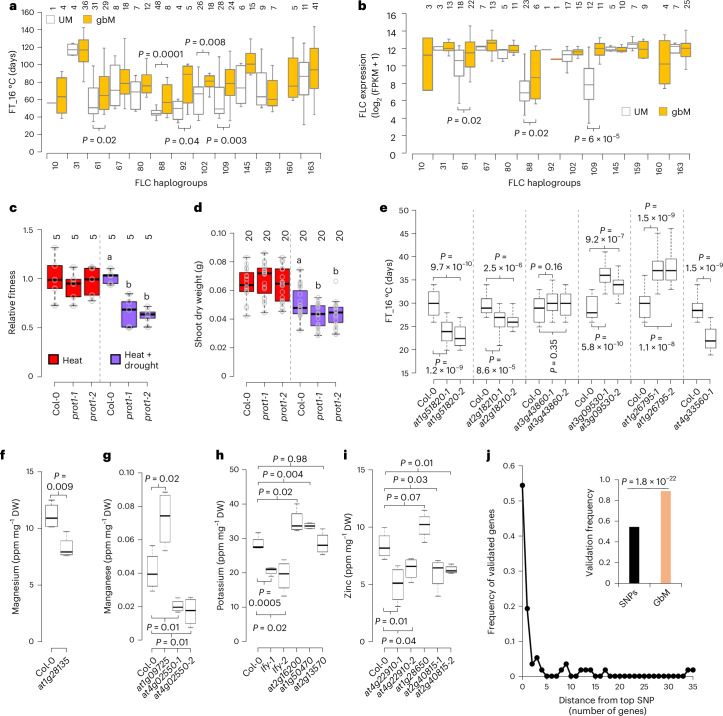
GbM variation enables efficient identification of new functional genes. **a**,**b**, Association of FLC epiallelic states with FT_16 °C (**a**) and FLC expression (**b**) in 13 FLC haplogroups invariant for SNPs and known SVs. Only accessions without TE polymorphism around FLC are considered. The number of accessions corresponding to each haplogroup are indicated. *P* values correspond to two-tailed Student’s *t*-test. **c**,**d**, Fitness (**c**) or shoot dry weight (**d**) of *prot1* mutants (two independent alleles) relative to Col-0 under heat or joint heat and drought stress. Numbers of independent experiments are indicated for fitness (**c**), and plant numbers are indicated for shoot weight (**d**). Different letters signify *P* < 0.05, one-way analysis of variance, Tukey’s test. **e**, Flowering time of Col-0 and knockout mutants of *AT1G51820*, *AT2G18210*, *AT3G43860*, *AT3G09530*, *AT1G26795* and *AT4G33560*. Plants were grown at 16 °C. *P* values were calculated using a two-tailed *t*-test. **f**–**i**, Magnesium (**f**), manganese (**g**), potassium (**h**) and zinc (**i**) levels of Col-0 and knockout mutants of *LEAFY*, *AT2G16200*, *AT1G50470*, *AT2G13570*, *AT4G22910*, *AT1G28650*, *AT2G40815*, *AT1G09725*, *AT4G02550* and *AT1G28135*. The number of biological replicates is 4. *P* values were calculated using a two-tailed *t*-test. Sample medians are represented by centre lines within the box plots (**a**–**i**). Box limits indicate the 25th and 75th percentiles; whiskers extend to 1.5 times the interquartile range. DW, dry weight. **j**, Distance (number of genes) of validated gene from the top SNP (SNP displaying the lowest association *P* value) in GWA analyses of various complex traits. Inset shows the frequency of top SNP located within the validated gene (encompassing the region from the end of the upstream gene to the beginning of the downstream gene) and the validation frequency for genes containing associated gbM variants. *P* values from the two-tailed Student’s *t*-test comparing the two groups are shown.

**Extended Data Fig. 7:**
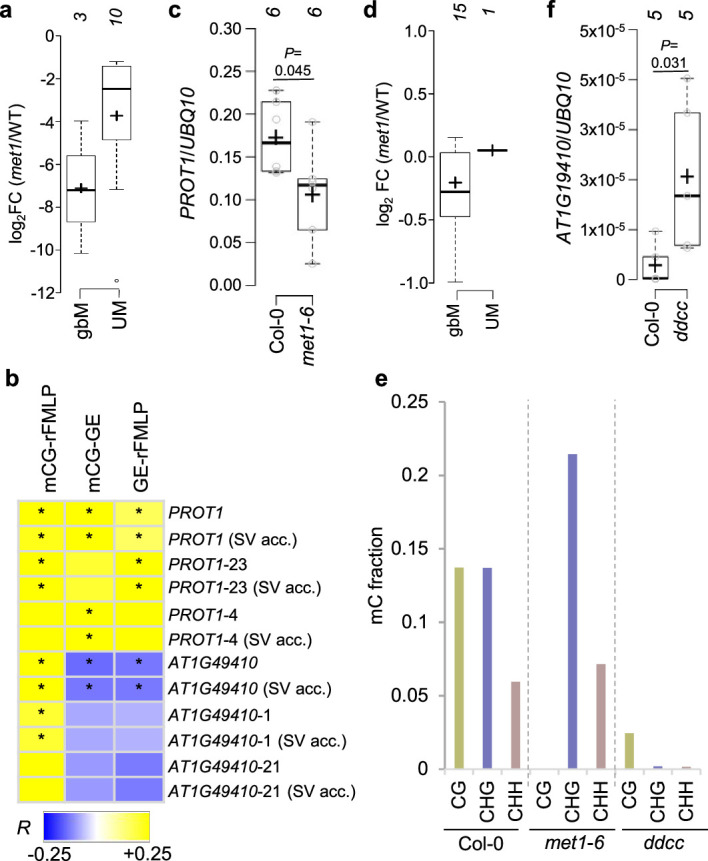
Methylation and expression of epigenetic flowering and relative fitness QTLs. (**a**) *FLC* expression change in *met1* lines of accessions with either gbM or unmethylated (UM) *FLC* epialleles in wild-type (WT), assessed by RNA-seq^75^. Numbers of accessions within each group are indicated. (**b**) Tripartite association between intragenic DNA methylation (mCG), gene expression (GE), and relative MLP fitness (rFMLP) in the entire population and after accounting (acc.) for structural variation (SV). Associations between the three variables are shown in two independent haplogroups of *PROT1* (PROT1-23 and PROT1-4) and *AT1G19410* (AT1G19410-1 and AT1G19410-21) before and after accounting for SV. *P < 0.05, Pearson’s correlation test. (**c**) Transcript levels of *PROT1* relative to *UBQ10* in Col-0 and *met1-6*, assessed by qRT-PCR in six biological replicates. *P*, two-tailed Student’s t-test. (**d**) *PROT1* expression change in *met1* lines of accessions with either gbM or UM *PROT1* epialleles in WT, assessed by RNA-seq^75^. Numbers of accessions within each group are indicated. (**e**) DRM and CMT methyltransferases control teM of *AT1G19410*. Fractional methylation in CG, CHG, and CHH sequence contexts is shown in indicated genotypes. (**f**) Transcript levels of *AT1G19410* (*ANH*) relative to *UBQ10* in Col-0 and *ddcc*, assessed by qRT-PCR in five biological replicates. *P*, two-tailed Student’s t-test. Center lines within box plots represent sample medians and plus signs correspond to means. Box limits indicate the 25th and 75th percentiles, whiskers extend to 1.5 times the interquartile range.

We focused special attention on *FLC* (QTL^gbM^) and the two MLP fitness QTLs—*Proline Transporter 1* (*PROT1*; *AT2G39890*; QTL^gbM^) and *AT1G19410* (QTL^teM^)—because we identified *FLC* and *PROT1* as +eQTL^gbM^ and *AT1G19410* as a −eQTL^teM^ (Supplementary Table 3). Because multiple *FLC* SNP and SV alleles affect flowering time or vernalization response^37,85,86^, we defined 13 *FLC* haplotypes that were invariant for SNPs and known SVs^74^ (Supplementary Table 16), 12 of which contain gbM and UM accessions (Fig. 4a), suggesting complex gbM evolution at this locus. GbM accessions display significantly delayed flowering (flowering time at 16 °C, FT_16 °C) within five haplotypes (delay of >18 days in three haplotypes; Fig. 4a), and significantly higher *FLC* expression in three of these haplotypes (Fig. 4b). These results suggest that gbM promotes *FLC* expression, as expected for a +eQTL^gbM^, and are consistent with the known function of *FLC* in delaying flowering^84^. Although upstream teM has been linked to *FLC* expression and flowering time^87^, exclusion of the relevant teM accessions does not alter our results, and in general we find that upstream teM is uncorrelated with *FLC* expression or flowering time (Supplementary Figs. 11–13). *FLC* is downregulated in *met1* regardless of WT methylation status (Extended Data Fig. 7a), suggesting indirect effects of global methylation loss.

For *PROT1* and *AT1G19410*, we found consistent associations between mCG, fitness, and expression after accounting for SV in the entire population, as well as in haplogroups invariant for SNPs and SVs (Extended Data Fig. 7b and Supplementary Table 16). As expected for a +eQTL^gbM^, *PROT1* is downregulated by 38% in *met1* as determined by quantitative reverse transcription PCR (qRT–PCR; Extended Data Fig. 7c and Supplementary Table 17) and is downregulated in *met1* RNA-seq data from accessions in which *PROT1* is methylated (Extended Data Fig. 7d). *AT1G19410* teM is lost in plants that lack DRM and CMT methyltransferases (Extended Data Fig. 7e), and in such *ddcc* mutants^88^ *AT1G19410* expression increases about sevenfold (Extended Data Fig. 7f and Supplementary Table 17), consistent with a −eQTL^teM^.

**Extended Data Fig. 8:**
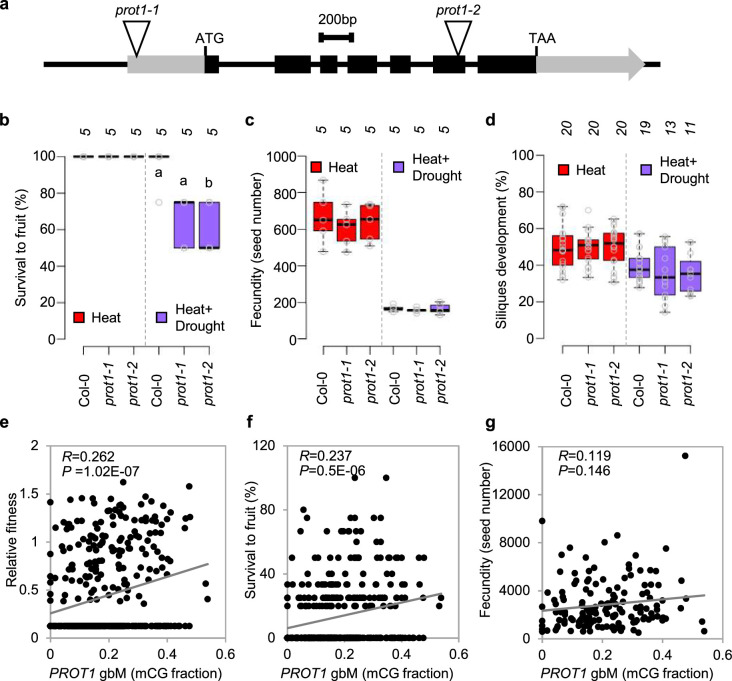
*PROT1* promotes fitness under heat and drought stress. (**a**) Schematic representation of *PROT1* genomic regions with positions of the T-DNA insertions. (**b**-**d**) Box plots showing survival to fruit (**b**), fecundity (**c**), and fertility (% of flowers developing siliques; **d**) phenotypes of *prot1* mutant and Col-0 wild type plants under heat stress (red) or combined heat and drought stress (purple). Numbers of independent experiments are indicated for survival to fruit (**b**) and fecundity (**c**). Numbers of plants are indicated for fertility (**d**). Box boundaries indicate the 25th and 75th percentiles, whiskers extend to 1.5 times the interquartile range, center lines correspond to medians. Different letters signify *P* < 0.05, one-way ANOVA, Tukey’s test. (**e**-**g**) Association of *PROT1* gbM with relative fitness (**e**), survival to fruit (**f**), and fecundity (**g**). Correlation coefficients (*R*) and *P* values of Pearson’s correlation test are indicated.

**Extended Data Fig. 9:**
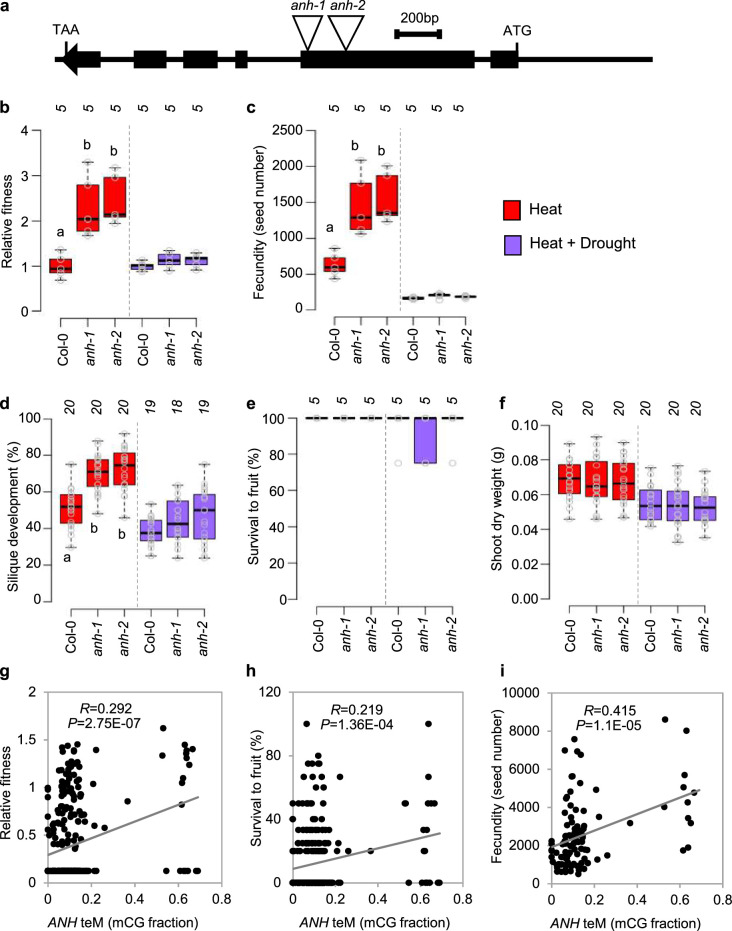
AT1G19410 (*ANH*) reduces fertility under heat stress. (**a**) Schematic representation of *ANH* genomic region with positions of the T-DNA insertions. (**b**-**f**) Box plots show relative fitness (**b**), fecundity (**c**), fertility (**d**), survival to fruit (**e**), and shoot dry weight (**f**) of *anh* mutants (two independent alleles) relative to Col-0 under heat stress (red) or joint heat and drought stress (purple). Numbers of independent experiments are indicated for relative fitness (**b**), fecundity (**c**), and survival to fruit (**e**). Numbers of plants are indicated for fertility (**d**) and shoot weight (**f**). Box boundaries indicate the 25th and 75th percentiles, whiskers extend to 1.5 times the interquartile range, center lines correspond to medians. Different letters signify *P* < 0.05, one-way ANOVA, Tukey’s test. (**g**-**i**) Association of *ANH* teM with relative fitness (**g**), survival to fruit (**h**), and fecundity (**i**). Correlation coefficients (*R*) and *P* values of Pearson’s correlation analysis are indicated.

The positive associations between fitness and mCG in *PROT1* and *AT1G19410* make clear predictions about the effects of gene inactivation: *PROT1* (+eQTL^gbM^) inactivation should reduce fitness, whereas *AT1G19410* (−eQTL^teM^) inactivation should enhance fitness. Genetic inactivation of *PROT1* indeed caused ~35% fitness reduction under joint heat and drought stress (Fig. 4c and Extended Data Fig. 8a). *PROT1*-mutant plants produced less biomass and had decreased survival to fruit, but had the same fecundity (seed set) as WT (Fig. 4d and Extended Data Fig. 8b–d). Consistently, *PROT1* gbM is specifically associated with survival in MLP conditions (Extended Data Fig. 8e–g). Inactivation of *AT1G19410* resulted in a slight (~13%) but non-significant increase in relative fitness under heat and drought stress (Extended Data Fig. 9a,b). However, *AT1G19410* mutants have greatly enhanced (>2-fold) fitness under heat stress alone, with >2-fold increased fecundity and significantly increased fertility (percentage of flowers developing siliques), but no major effect on survival or biomass (Extended Data Fig. 9b–f). Therefore, we named *AT1G19410 ANAHITA* (*ANH*) after the ancient Persian goddess of fertility and water. Notably, the association of *ANH* teM is stronger with fecundity than survival in MLP conditions (Extended Data Fig. 9g–i). Thus, although both genes influence relative fitness, *PROT1* specifically influences survival, whereas *ANH* affects fecundity.

To more broadly examine the validity of epiGWA mapping, we analysed the six additional flowering time QTL^gbM^ genes, and ten QTL^gbM^ genes associated with accumulation of the most easily quantifiable minerals—potassium (K), magnesium (Mg), manganese (Mn) and zinc (Zn)—using T-DNA insertion mutants. We focused on gbM QTLs because these are much more numerous and because gbM variation is unambiguously epigenetic. Mutants in all flowering time QTL^gbM^ genes except *AT3G43860* showed significantly altered FT_16 °C (Fig. 4e), and mutants in nine mineral QTL^gbM^ genes displayed significant changes in the accumulation of relevant minerals (*P* ≤ 0.07, eight genes with *P* ≤ 0.03; Fig. 4f–i). Thus, we validated nearly 90% (16/18, including the published *flc* flowering phenotype^89^) of QTL^gbM^ via mutations in genes where gbM is associated with the trait. A comparative analysis of *Arabidopsis* SNP-based GWA studies across 48 diverse traits with 57 validated genes (Supplementary Table 18) revealed that the SNP with the lowest *P* value is located within the validated gene in only ~54% of cases (Fig. 4j). The high frequency of epiGWA pinpointing the trait-relevant gene is probably due to gbM epimutation rates exceeding genetic mutation rates by ~10^5^-fold^66,90–92^. Such turnover should rapidly disrupt linkage between gbM polymorphism, so that only gbM in the causative gene is associated with trait variance. Given that the associations obtained with GWA and epiGWA analyses rarely overlap (Supplementary Figs. 6, 9 and 10), gbM-based epiGWA mapping presents a powerful and broadly applicable gene discovery tool, as we illustrate by identifying 15 new genes affecting six distinct phenotypes (MLP fitness, flowering time and accumulation of K, Mg, Mn and Zn).

### GbM variation may facilitate local adaptation

**Fig. 5:**
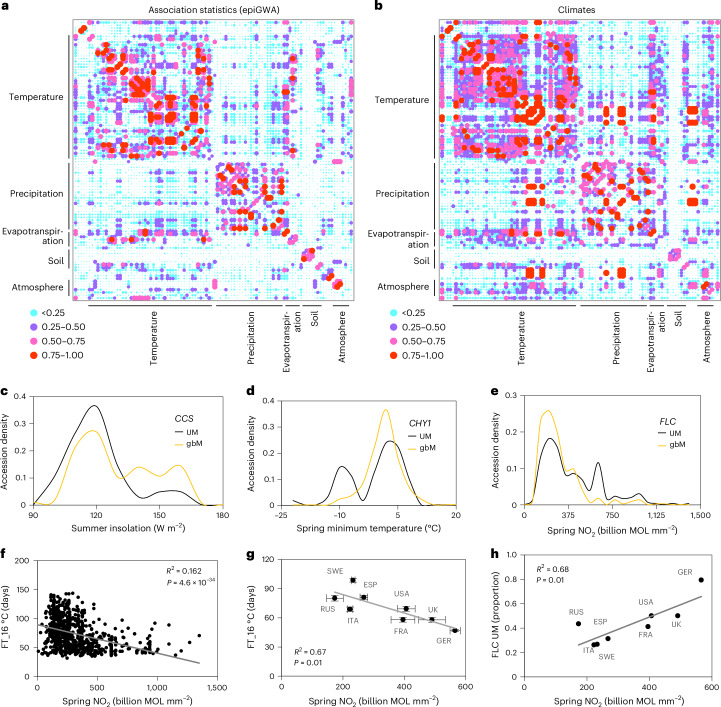
GbM variation is associated with geoclimatic variables*.* **a**,**b**, Correlation (*R*^2^) matrices of epiGWA *P* values (**a**) and environmental variables (**b**) for 57 genes identified in at least three epiGWA analyses. Associations between epiallelic states (UM and gbM) of genes and environmental variables were examined using a mixed linear model. Supplementary Tables 20 and 21 list individual environment labels in order. **c**–**e**, Associations between gbM and environmental data for *CCS* (**c**), *CHY1* (**d**) and *FLC* (**e**). **f**, Pearson’s correlation between springtime atmospheric NO_2_ (billion molecules (MOL) per mm^2^) and flowering time (FT_16 °C) of individual accessions. **g**, Average (± s.e.m.) FT_16 °C and NO_2_ concentrations in Sweden (SWE, number of accessions (*N*) = 187), Russia (RUS, *N* = 47), Italy (ITA, *N* = 48), Spain (ESP, *N* = 170), USA (*N* = 41), France (FRA, *N* = 37), UK (*N* = 56) and Germany (GER, *N* = 102). **h**, Prevalence of *FLC* UM epiallele as a function of NO_2_. *R*^2^ and *P* values indicated in **g** and **h** are derived from Pearson’s correlation test.

**Extended Data Fig. 10:**
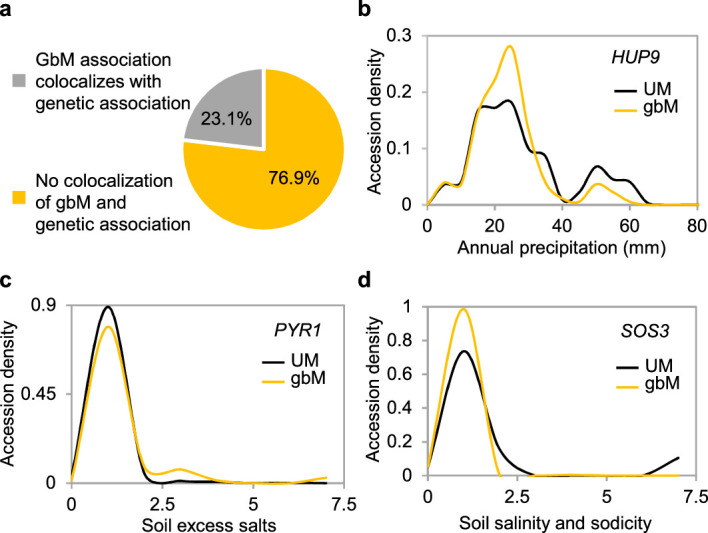
Associations between gbM variation and the environment. (**a**) Frequency of associations between gbM and environmental data colocalizing with genetic associations. (**b**-**d**) Associations between gbM and environmental data for *HUP9* (**b**), *PYR1* (**c**), and *SOS3* (**d**).

*Arabidopsis* grows in a broad range of natural environments and shows extensive local adaptation^93^. As we find that gbM polymorphism explains substantial gene expression variation, we tested whether gbM may facilitate adaptation by performing epiGWA analyses for 171 environmental variables^94^. We detected 571 associations between 232 genes and 115 of these variables, with 77% of these associations not colocalizing with SNP associations (Extended Data Fig. 10a and Supplementary Table 19). Notably, gbM variation in 57 genes is associated with at least three environments, and *P* values for these genes are strongly correlated for associated environments (Fig. 5a,b and Supplementary Tables 19–21), suggesting that multiple correlated environmental conditions impose selection on epiallelic states of individual genes.

Our analysis identified several notable gbM associations with a plausible functional link to environmental adaptation (Fig. 5c–e and Extended Data Fig. 10b–d) that do not overlap with genetic associations (Supplementary Table 19). GbM variation in *CCS*, which mediates heat stress responses^95^, is associated with summer insolation, with gbM epialleles more prevalent in high insolation environments (Fig. 5c). GbM in *CHY1*, which is involved in cold signalling and promotes freezing tolerance^96^, is associated with spring minimum temperature, with gbM epialleles rare in environments where temperature drops below −4 °C (Fig. 5d). GbM in *HUP9*, a regulator of flooding stress response^97^, is associated with annual precipitation (Extended Data Fig. 10b). *PYR1* gbM variation is associated with soil excess salts, with high-salt soils almost exclusively featuring gbM epialleles (Extended Data Fig. 10c). *PYR1* is an abscisic acid receptor^98^, and abscisic acid is a central regulator of plant salt stress responses^99^. GbM variation in the calcium sensor *SOS3*^100^ associates with soil salinity and sodicity (Extended Data Fig. 10d), which includes calcium carbonate (CaCO_3_) and gypsum (CaSO_4_·2H_2_O). Nearly all accessions from high-salinity and high-sodicity soils have UM *SOS3* epialleles (Extended Data Fig. 10d). These findings suggest that natural gbM variation facilitates local adaptation in native habitats.

The most striking association we discovered is between *FLC* gbM and springtime concentration of nitrogen dioxide (NO_2_), with UM *FLC* alleles prevalent in high-NO_2_ environments (Fig. 5e and Supplementary Table 22). Because UM *FLC* accessions flower early (Fig. 4a), this association predicts that accessions from high-NO_2_ environments should flower early. Indeed, flowering time (FT_16 °C) of laboratory grown *Arabidopsis* accessions is more strongly correlated with atmospheric NO_2_ in native environments than with any other environmental variable (Fig. 5f and Supplementary Table 23). NO_2_ levels vary regionally and are indicative of air quality in urban and industrial centres^101^. We find that average concentrations of NO_2_ across countries show a remarkable linear correlation (*R*^2^ = 0.67) with flowering time in the laboratory (Fig. 5g), suggesting that earlier flowering is advantageous in higher-NO_2_ environments. Prevalence of the *FLC* UM epiallele in countries is also strongly correlated with NO_2_ (*R*^2^ = 0.68; Fig. 5h). These findings suggest *FLC* gbM variation is selected to adapt flowering time to atmospheric NO_2_ (or an unevaluated correlated environmental factor).

## Discussion

Our findings reveal that gbM and teM are independent phenomena (Fig. 1) that explain substantial amounts of gene expression variation in the *Arabidopsis* population (Fig. 2). GbM is most important for broadly and constitutively expressed genes (Fig. 2f,h), consistent with its enrichment in such genes^67,68^, whereas teM is most relevant for genes with narrow or inducible expression (Fig. 2e,g). We also find that gbM directly and quantitatively affects gene expression (Fig. 3), and that its natural variation can be used to identify many new genes that influence a range of complex traits (Fig. 4). There is a great deal of gbM variation: just the core gbM genes analysed in Fig. 1e contain 299,679 polymorphic CG sites, compared with the 920,998 common SNPs across the *Arabidopsis* genome used in our analysis (Fig. 2). Thus—as for SNPs—many small effects can accumulate within gene networks to substantially influence gene expression (Fig. 2a–c). Overall, our results indicate that epigenetically variable gbM patterns are a major source of functional polymorphism in *Arabidopsis*.

Because DNA methylation is mutagenic^102^, and its presence in coding sequences probably incurs a fitness cost^45^, the widespread conservation of gbM in plants and animals has presented a mystery. A potential explanation is that gbM variation can rapidly generate a range of gene expression epialleles, thereby accelerating adaptation to new or changing environments. The association between atmospheric NO_2_, flowering time and *FLC* gbM (Fig. 5e–h) presents an illustration of how this might occur. Natural genetic variation at *FLC* is a major determinant of flowering time^82,85,86^ and is associated with over 20 environmental variables that are (or may plausibly be) related to flowering, including latitude, temperature and precipitation, but not NO_2_ (ref. ^94^). The majority of atmospheric NO_2_ (>75%) is produced by recent human activity, especially the burning of fossil fuel^103^. Therefore, *Arabidopsis* populations have had to adapt to NO_2_ concentrations (or a correlated unexamined environmental variable) changing over a few decades. Genetic adaptation at *FLC* apparently has not yet occurred in response to such rapid environmental alteration, or at least is too weak for detection. However, epigenetic gbM variation at *FLC* is significantly associated with atmospheric NO_2_ (Fig. 5e–h), but not other environmental variables (Supplementary Table 22), which is consistent with our observation that *FLC* gbM and sequence variation are independent (Fig. 4a). Therefore, gbM variation at *FLC* has probably facilitated adaptation to anthropogenic NO_2_ increases, whereas genetic variation has been involved in adaptation to environmental conditions that vary over longer timescales. This interplay between epigenetic and genetic adaptation is consistent with evolutionary models^9–11^ and may be a generally important component of environmental adaptation.

## Methods

### Methyl-C seq data analysis

Bisulfite sequence reads were accessed for the 1001 methylomes^40^ experiments from the Sequence Read Archive (SRA) under accession number GSE43857. Sequencing reads of 948 non-redundant *Arabidopsis* accessions were aligned to the *Arabidopsis* TAIR10 genome reference sequence^104^, using BSMAP^105^ with default parameters, and known SNPs and indels^82^ were masked. Genes and transposons were annotated using the Araport11 annotation^106^. Methylomes were segmented into UM, gbM and teM segments as previously described^54^. The result of this segmentation is that gbM segments contain mCG anywhere between the annotated transcriptional start and termination sites of genes (and can span exons and/or introns) and lack non-CG methylation, teM segments contain non-CG methylation and UM segments lack methylation. Methylation of each CG site was called by comparing the counts of aligned reads indicating methylated and unmethylated status at the site. Fisher’s exact test was used to determine whether there was sufficient read coverage at the site to distinguish the site from a fully unmethylated site with an error rate similar to the methylation rate observed in the chloroplast of the sample in question (as an estimate of bisulfite conversion inefficiency), or from a fully methylated site with a similar error rate. For sites where these tests indicated coverage was sufficient, a binomial test was used to identify sites with significantly more methylated reads than expected at an unmethylated site. Sites with significantly more methylated reads than would be expected for an unmethylated site, but with less than 45% reads methylated, were classified as partially methylated and generally treated as missing data. A gene was classified as gbM, teM, both (gbM and teM), UM or indeterminate in each accession, based on overlapping methylome segments. Genes overlapped by a gbM segment three or more CG sites long, with at least one CG site called methylated by a binomial test, were classed as gbM genes, unless they are also overlapped by a teM segment at least 25% as long as the gbM segment, in which case they were classified as both. Genes overlapped by a teM segment three or more CG sites long were classified as teM genes, unless they are also overlapped by a gbM segment at least 25% as long as the teM segment, in which case they were classified as both. Genes not overlapped by gbM or teM segments and that span at least three sites called unmethylated by a binomial test were classified as UM. The remainder of genes were classified as indeterminate. Ambiguous genes (classed as ‘both’ or ‘indeterminate’) were discarded from further analysis. The mean CG methylation level of gbM or teM genes was calculated for each gene by summing the number of CG sites identified as methylated and dividing by the total number of CG sites classified as either methylated or unmethylated, as determined by a binomial test.

### Estimation of prevalence of teM across gbM conservation bins

The number of genes having gbM or teM epigenetic states was determined in 948 *Arabidopsis* accessions. Pearson’s correlation analysis for the number of gbM and teM genes was performed using accessions with more than 60% sequencing coverage of genomes. Conservation of epiallelic states of genes was analysed as a fraction of accessions having gbM or teM and the total available calls (that is, excluding accessions where the gene could not be called). Average prevalence of teM within gbM conservation bins was estimated in four gbM categories (0; >0% but <10%; 10–90%; and >90%), decile gbM bins and percentile gbM bins. To compare our results with published findings, identical analyses were performed using available data^60^ with restrictive definitions of gbM and teM.

### Methylation level distribution

Simulation of steady-state gbM was previously described^66^. In brief, genic regions were refined by excluding sequences not methylated in the population or containing high levels of histone H2A.Z, which is known to antagonize DNA methylation^107^. This resulted in a single, continuous methylatable region per gene for 7,980 genes^66^. Further stringent filtering removed genes with a methylatable region covering less than 80% of the annotated gbM segment, refining the dataset to 6,736 genes. GbM within these loci was simulated from an entirely unmethylated starting state for 100,000 generations^66^. To ensure robust comparison with natural variation, 740 iterations of the simulation were performed to produce a distribution of gbM levels for comparison with the empirical distribution over 740 accessions with global gbM levels similar to Col-0^66^. Loci were grouped into percentiles by their gbM conservation level, with multiple data points for each gene showing mCG levels in different accessions or simulation iterations.

### Partitioning expression variance attribution between gbM, teM and SNPs

RNA-seq data for 625 *Arabidopsis* accessions were retrieved from Gene Expression Omnibus (GEO): GSE80744 (ref. ^1^). Genes without detectable expression in leaves of >50% of accessions were discarded. To avoid confounding by low allele frequencies, we selected gbM and teM genes having at least one mCG site in >20% of accessions. This yielded a set of 10,206 genes with gbM polymorphism and 1,442 genes with teM polymorphism. From the imputation version of the 1001 genome SNP panel^4^, we selected common SNPs (frequency 15% and above), giving 920,998 SNPs. We then modelled the expression of each gene, ${y}_{j}$ (a vector of length 625 accessions), as dependent upon the joint effects of gbM, ${X}_{{{\mathrm{gbM}}}}$ (a matrix with 625 rows and 10,206 columns), teM, ${X}_{{{\mathrm{teM}}}}$ (a matrix with 625 rows and 1,442 columns) and the SNPs, ${X}_{{{\mathrm{snps}}}}$ (a matrix with 625 rows and 920,998 columns), with the model

$${{y}}_{j}={\text{X}}_{{{\mathrm{gbM}}}}{{b}}_{{{\mathrm{gbM}}}}+{{X}}_{{{\mathrm{teM}}}}{{b}}_{{{\mathrm{teM}}}}+{{X}}_{{{\mathrm{snps}}}}{{b}}_{{{\mathrm{snps}}}}+\epsilon,$$

where ${b}_{{{\mathrm{gbM}}}},{b}_{{{\mathrm{teM}}}}$ and ${b}_{{{\mathrm{snps}}}}$ are regression coefficient vectors of length 10,206, 1,442 and 920,998 of the jointly estimated effects of gbM, teM and the SNPs, respectively, on the expression values of gene *j*. Each regression coefficient is modelled as coming from a mixture of normal distributions and a Dirac delta spike at zero. We fit this model using software for methylation data analysis that has been used extensively in human studies^70^. GbM, teM and SNP effects are modelled as three independent groups with independent priors, where the total phenotypic variance attributable to each component is estimated from the data. Note that, while the groups have independent priors, each effect is modelled conditional on all other effects in the same group and all other groups. Altogether, we modelled 14,000 genes (genes need not have *cis* gbM or teM variance to be modelled, as the expression of each gene is modelled using the entire set of gbM, teM and SNPs). We checked convergence of the parameters across 5,000 posterior samples, discarding genes for which the analysis was highly divergent and retaining those (7,339; Supplementary Table 1) for which all parameters were estimated in a stable manner that was repeatable across multiple runs of the algorithm. Frequency distributions of the partitioned expression variance were generated via the kernel density estimation function in R.

### Associations of intragenic DNA methylation with gene expression levels

RNA-seq data for 625 *Arabidopsis* accessions with gene-specific mCG levels were retrieved from GEO: GSE80744 (ref. ^40^). Genes showing no detectable expression in leaves of any of these accessions were discarded from association analyses. Furthermore, to avoid confounding by low allele frequencies, these analyses were performed using gbM and teM genes having at least one mCG site in more than 10% *Arabidopsis* accessions. This allowed us to examine associations between mCG levels and gene expression for 18,679 gbM and 1,442 teM genes. Expression levels of genes were regressed on mCG levels in a linear model. Association *P* values for Pearson correlation were estimated using SigmaPlot 14.0.

Bonferroni (*α* = 0.05) or 0.05 and 0.1 false discovery rate^108^ (FDR) corrections were implemented to account for multiple tests. The percentage of expression variance explained by intragenic DNA methylation was calculated as

$${\rm{PVE}}=\frac{{(\;\beta )}^{2}(V_{\mathrm{mCG}})}{V_{\mathrm{P}}},$$

where *V*_mCG_ is the variance of mCG, *V*_P_ corresponds to phenotypic (expression) variance and *β* effects for each association test were calculated as

$$\beta =R\,{{x}}\left(\frac{\sigma_{\mathrm{P}}}{\sigma_{\mathrm{mCG}}}\right)$$

where *R* is Pearson’s correlation coefficient, *σ*_P_ corresponds to standard deviation of gene expression and *σ*_mCG_ is standard deviation of mCG in the population.

### Gene feature annotation

CG (CGG or CGT or CGC or CGA) sites were enumerated by scanning annotated genes^106^ within the Col-0 reference sequence^104^ with a three-base window and step size of one base. Gene lengths were obtained from the Col-0 annotation^106^. Then, CG dinucleotide frequencies were calculated by normalizing the number of CG sites to a gene’s annotated length. The mean expression level of each gene was calculated across 625 accessions. Shannon entropy data for 25,707 genes^109^, ancestral genic methylation states^51^, and H3K9me2 and non-CG methylation data for *met1*-mutant plants compared with WT^78^ were obtained from published sources.

### Pipeline to account for SNP effects on the expression of eQTL^gbM/teM^ genes

To disentangle the effects of intragenic methylation on expression from *cis*-acting DNA sequence changes, we performed GWA analyses for the expression of 765 eQTL^gbM^ and 217 eQTL^teM^ Bonferroni genes using 1001 genomes SNP^82^ data in an accelerated mixed model^110^. Colocalization of each *cis* eQTL (eQTL^SNP^) significant at Bonferroni threshold (*α* = 0.05) with epigenetic eQTL was determined. The eQTL^gbM/teM^ genes for which no colocalized *cis* eQTL^SNP^ were detected are considered to affect gene expression variation independently of genetic variation (retained eQTL^gbM/teM^) (Extended Data Fig. 4a,b and Supplementary Fig. 1). In cases where eQTLs^gbM/teM^ colocalized with eQTLs^SNP^, the original population of accessions was separated into two nested populations, each fixed for the GWA SNP (Supplementary Fig. 1). Associations between intragenic DNA methylation and expression of these genes were reexamined within nested populations to account for the effects of SNP variation on expression. The genes that exhibited significant association between intragenic DNA methylation and expression in at least one nested population were also classified as retained eQTL^gbM/teM^. Genes without significant associations between intragenic DNA methylation and gene expression in nested populations were considered probably confounded by linked SNPs in the population. Accordingly, these eQTL^gbM/teM^ were classified as lost eQTL^gbM/teM^ genes. To account for GWA SNP effects on expression variance, the per cent variance explained by methylation was calculated in nested populations as described above.

### Analysis of published *met1* RNA-seq data

RNA-seq data for *met1* mutants were retrieved from PRJEB54036 (ref. ^75^) for 16 different accessions of *Arabidopsis* (Aa-0, Baa-1, Bs-1, Bu-0, Col-0, Com-1, Cvi-0, Ei-2, Est-1, MAR2-3, Nok-3, Pi-0, Ste-0, Tscha-1, Tsu-0 and Uk-1). Reads were mapped to the genome using HiSat2, and changes in expression in comparison with WT across annotated genes (Araport11) identified using feature counts and DESeq2^111^. Independent alleles of *met1* were analysed separately. Variability of these samples was calculated using the coefficient of variation of the TPM across three biological replicates separately for *WT* and *met1*. Only genes with detected reads in all biological replicates were used. Genes with no change in expression were additionally identified using DESeq2, selecting genes with an adjusted *P* value >0.05 and log_2_ expression change between −1 and 1. Methylation levels for these accessions were extracted from the 1001 methylomes dataset^40^, and gbM genes with mCG >5% spanning the transcription start site between −100 bp and 250 bp were excluded from expression analyses. Additional Col-0 datasets^54,77^ were retrieved from GSE93584 and GSE122394 for inflorescence, leaf and seedling, then aligned, and log_2_FC was calculated as above.

### Haplotype analyses

To account for allelic heterogeneity, associations between methylation and expression were examined within haplotypes. SNPs within and 4 kb upstream and downstream of genes were extracted from an imputed version of the 1001 genome SNP panel^82^. Sequences were aligned, and the accessions invariant for SNPs over the entire region for each gene were classified into a haplogroup. Haplogroups comprising fewer than 15 accessions were discarded from association analyses. Associations of mCG with gene expression or phenotypes were examined within haplogroups to fully account for the effects of local SNP variation on expression or phenotypic variation.

### Accounting for SV effects on epigenetic QTLs

Structural variants were identified within epigenetic QTLs and 4 kb upstream and downstream using published TE polymorphism data in *Arabidopsis* accessions^74^. Associations between structural polymorphism and expression were examined using a linear model and the effects of structural variants on epigenetic QTLs were accounted through analysis in populations invariant for TE polymorphism^74^.

### EpiGWA studies for relative fitness

EpiGWA analyses for relative fitness were performed using published relative fitness data^81^ of 412 *Arabidopsis* accessions with sufficient mCG information. Common garden experiments had been performed in two climatically distinct field stations in Madrid (M) and Tübingen (T)^81^. Madrid presents a climate that transitions between Mediterranean and semi-arid climates and Tübingen is characterized by a temperate climate with no dry season and warm summers. High (H) and low (L) rainfall conditions typical of Tübingen and Madrid had been simulated during these experiments. To mimic low- and high-density populations in nature, individual (I) or multiple plants (P) had been grown in pots. EpiGWA analyses were performed using a linear model to assess associations between gbM or teM levels of genes and relative fitness. For these analyses, we focused on genes having gbM or teM conserved in more than 10% of *Arabidopsis* accessions. Linear model association mapping analyses may detect excessive significant marker-trait associations due to underlying population structure^112^. We, however, detected only two associations (*PROT1* and AT1G19410) at 0.05 FDR for relative fitness in MLP (Supplementary Table 8). In addition, gbM variation in one gene *MuDR* (AT1G64255) is associated with relative fitness in MLI at 0.1 FDR. We next used quantile–quantile (QQ) plots and genomic control inflation factor *λ* (ref. ^113^) to assess confounding of association statistics (Supplementary Fig. 5 and Supplementary Table 7). *λ* was calculated using unlinked markers as

$$\lambda =\frac{{\mathrm{Median}}\,{X}^{2}\,{\mathrm{observed}}\,P}{{\mathrm{Median}}\,{X}^{\;2}\,{{\mathrm{expected}}}\,{P}},$$

where *X*^2^ is the chi-square and *P* is the *P* value.

*λ* varied between phenotypes and ranged from 0.91 (relative fitness MHP (Madrid, High rainfall conditions, multiple Plants per pot)) to 1.48 (relative fitness TLI (Tübingen, Low rainfall conditions, Individual plants per pot)) (Supplementary Table 7). To control for confounding effects of population stratification, association statistics were corrected using *λ*, and the genome-wide significance threshold was recalculated using corrected *P* values. Both *PROT1* and AT1G19410 associations were significant at 0.05 FDR; however, *MuDR* was not significant at 0.1 FDR. Associations between intragenic DNA methylation and fitness significant at 0.05 FDR^108^ are called epigenetic QTLs in this study. Tripartite associations between mCG levels, gene expression and relative fitness in MLP for *PROT1* and AT1G19410 were analysed using a linear model.

### EpiGWA studies for flowering-related traits

Three types of epiGWA mapping were performed for flowering-related traits to identify the best model to account for confounding effects of population structure. A linear model was employed using mCG levels of genes, and two models, a generalized linear model (GLM) and a mixed linear model (MLM), were used for epiGWA using epiallelic states (UM or gbM; UM or teM) of genes. The methods for determination of epiallelic states of genes are described in the ‘Methyl-C seq data analysis’ section. The numbers of *Arabidopsis* accessions used for these epiGWA analyses are listed in Supplementary Table 9.

Linear model epiGWA mapping was performed to examine associations between mCG levels of genes (>10% gbM or teM conservation) and flowering time data (flowering time at 10 °C (FT_10 °C) and 16 °C (FT_16 °C))^82^. Association statistics for these epiGWA analyses were highly confounded (*λ* = 4.50 for FT_10 **°**C and λ = 4.52 for FT_16 **°**C; Supplementary Fig. 7 and Supplementary Table 10). Around 7,500 genes showed significant associations between mCG levels and flowering time at 0.05 FDR (Supplementary Fig. 7). Applying uniform *λ* correction for association *P* values in such cases is unsatisfactory for correcting population structure at genes with strong differences in mCG levels across subpopulations and can also result in a loss of statistical power at genes with uniformly distributed mCG levels^114,115^. Given the correlation of flowering with geographic regions, similar confounding of association statistics has been reported for flowering-related traits in *Arabidopsis* GWA studies^112^. Strong confounding of *P* values renders linear model epiGWA using mCG levels inappropriate for association mapping in structured populations.

Next, we used binary epiallelic states of genes to perform GLM and MLM epiGWA mapping using FT_10 °C and FT_16 °C flowering time phenotypes and seven additional flowering-related phenotypes^116^ (number of days for inflorescence stalk to reach 1 cm, number of days to the opening of first flower, number of cauline leaves, number of rosette leaves, cauline branch number, primary number of inflorescence branches and length of primary inflorescence stalk). GLM implemented in TASSEL^117^ is a fixed-effects linear model that we used to test associations between epiallelic states and phenotypes. Association *P* values for several of the flowering phenotypes deviated significantly from expected distribution of *P* values, as indicated by QQ plots and *λ* estimates (Supplementary Fig. 8 and Supplementary Table 11). Hence, GLM using epiallelic states is also inappropriate for epiGWA mapping in structured populations. Next, an MLM^117^ that includes both fixed and random effects was used to correct population structure. MLM can be presented as

$$Y=\beta X+Zu+e,$$

where *Y* represents the vector of phenotypes, *β* denotes the vector containing fixed effects including genetic markers and population structure (*Q* matrix), *u* captures variance due to relatedness between individuals (kinship (*K*) matrix), *X* and *Z* are the design matrices and *e* captures variance due to the environment. The *Q* matrix of population membership estimates was derived from principal component analysis of epiallelic states. The *K* matrix accounts for epigenome-wide patterns of relatedness between the individuals and was estimated using the identity-by-state method^117^. QQ plots and *λ* estimates based on MLM epiGWA showed no significant deviation of distribution of association *P* values from null distributions (Supplementary Fig. 8 and Supplementary Table 11). MLM was thus used to dissect the epigenetic architecture of flowering-related phenotypes. Genes having methylation calls in <10% accessions were removed.

The association between epiallelic states and expression levels of eight flowering epiQTL genes was analysed using MLM epiGWA mapping. To examine associations between gene expression and phenotypes, flowering phenotypes were regressed on quantitative variation of gene expression in a linear model. Associations between epiallelic states and flowering or gene expression phenotypes in nested populations were tested using MLM epiGWA analyses.

### EpiGWA studies for leaf mineral accumulation

Data for accumulation levels of 18 mineral elements^83^ in leaves of 934 *Arabidopsis* accessions were used for epiGWA analyses to identify gbM and teM variants associated with the diversity of these traits. EpiGWA analyses were performed using MLM implemented in Tassel^117^ as described above. We filtered out rare (minor allele frequency (MAF) <5%) gbM and teM variants. FDR 0.05 correction^108^ was implemented to account for multiple tests and identify significant associations.

### EpiGWA studies for geoclimatic variables

Data for 171 geoclimatic variables^94^ were used for epiGWA analyses to identify gbM variants associated with environmental variation in the native range of *Arabidopsis* accessions. EpiGWA analyses were performed using MLM implemented in Tassel^117^ as described above. We filtered out rare (MAF <5%) gbM variants. FDR 0.05 correction^108^ was implemented to account for multiple tests and identify significant associations. The density and distribution of *FLC*, *CHY1*, *CCS, HUP9*, *SOS3* and *PYR1* UM and gbM accessions was determined across the range of environmental variables.

### Genome-wide association studies for relative fitness, flowering and mineral phenotypes

GWA analyses were performed for relative fitness in eight climates^81^, nine flowering-related phenotypes^82,116^ and levels of 18 minerals^83^ using the same accessions as for epiGWA analyses. GWA mapping was carried out using 1001 genomes SNP data^82^ with an accelerated mixed model^110^ implemented in PyGWAS, a Python library for running GWAS (version 1.7.4). The accelerated mixed model has been shown to work well in previous studies for flowering and other phenotypes^14,110,118^. SNPs with MAF >5% in the population were considered. An FDR correction of 0.05 (ref. ^108^) was implemented to account for multiple tests and identify genetic QTLs.

### Genome-wide association to account for effects of *trans* QTLs on methylation variation

GWA analyses were performed for mCG levels of retained Bonferroni eQTL^gbM/teM^. GWA mapping was carried out as described above to identify *trans* genetic QTLs that are significant at the Bonferroni threshold. These analyses were performed in three *Arabidopsis* populations: worldwide populations that we used for association mapping for gene expression and phenotypes, 133 accessions of the Swedish panel, in which strong *trans* effects were found for around 1,300 gbM genes^50^, and a random non-Swedish worldwide population of equal size to the Swedish panel (Extended Data Fig. 5d–f). The percentage of mCG or epigenetic state variance explained by *trans* genetic QTLs was estimated as the ratio of sum of square of SNP markers (after fitting all other model terms) to the total sum of squares. If we consider only the 133 Swedish accessions, we find strong *trans* effects, with on average 37.9% of gbM variance explained at 11.5% of eQTL^gbM^ (4.4% gbM variance explained overall; Extended Data Fig. 5d–f). However, when we consider all 625 worldwide accessions, these *trans* effects nearly disappear; 9.7% of genes have significant *trans* QTLs, which on average explain 10.5% of gbM variance, with *trans* genetic variation accounting for only 1% of gbM variance over all tested eQTL^gbM^ (Extended Data Fig. 5). Notably, a panel of 133 randomly chosen worldwide accessions (same size as the Swedish panel) produced results that are almost identical to those of the Swedish panel and significantly different from the entire worldwide panel (Extended Data Fig. 5d–f). This indicates that estimates of *trans* effects on gbM variation are inflated in analyses of small populations, a phenomenon known as the Beavis effect^119,120^.

### RNA and bisulfite sequencing analysis of *h1* and *h1met1* mutants

Total RNA was extracted from 4-week-old *h1*^*−/−*^ and *h1*^*−/−*^*;met*^+*/−*^ leaves using Trizol (Invitrogen, cat. no. 15596026). To remove genomic DNA (gDNA) from samples, 1 mg of RNA was treated with the DNA-free DNA removal kit (Thermo, AM1907). Then, 100 ng of gDNA-depleted total RNA was used to construct RNA-seq libraries with Ovation RNA-seq systems 1–16 for the model organism *Arabidopsis* (Nugen, cat. no. 0351). To investigate the association of intragenic DNA methylation with expression level in *h1*^*−/−*^;*met1*^+*/−*^ plants, we first defined demethylated gbM genes as ones with more than 10% CG methylation, lose more than 5% CG methylation in *h1*^*−/−*^*;met1*^+*/−*^ versus *h1*^*−/−*^ plants and have less than 5% CG methylation in *h1*^*−/−*^*;met1*^+*/−*^. The gene expression fold change in *h1*^*−/−*^*;met1*^+*/−*^ plants (versus *h1*^*−/−*^ plants) was calculated using DeSeq2^111^. To analyse the association between gene expression and gbM change, we compared the average expression fold change of demethylated gbM genes and gbM genes that retain intragenic DNA methylation *in h1*^*−/−*^*;met1*^+*/−*^ plants.

For *h1*^*−/−*^;*met1*^+/+^ plants isolated from segregating *h1*^*−/−*^;*met1*^+*/−*^, 100–700 ng of DNA-depleted leaf RNA was used to construct RNA-seq libraries (Illumina, cat. no. 20020610 and 20019792) following the manufacturer’s manual. As segregating plants showed aberrant non-CG hypermethylation over gbM genes, we filtered out genes that gain non-CG methylation (average mCHG or mCHH>0.01). GbM genes that either lose or keep methylation were identified as described for *h1*^*−/−*^*;met1*^+*/−*^.

For bisulfite sequencing analysis of *h1*^*−/−*^;*met1*^+/+^ plants, we extracted gDNA from 4–5-week-old plant leaves. Then, 500 ng gDNA was sheared to 100–1,000 bp using Bioruptor Pico (Diagenode). gDNA libraries were constructed using NEBNext Ultra II DNA library prep kit for Illumina (New England Biolabs, cat. no. E7645). We performed bisulfite conversion twice (QIAGEN, cat. no. 59104) with ligated libraries and amplified libraries by PCR. Sequenced reads were mapped with the bs-sequel pipeline (https://zilbermanlab.net/tools/).

RNA-seq and DNA methylation data are deposited in GEO with accession GSE183785.

### Quantitative real-time PCR

Transcript levels of *PROT1* were quantified in Col-0 and *met1-6*^107^ with plants grown in a chamber with cycles of 16 h light (120 µE m^−2^ s^−1^) at 27 °C day and 16 °C night temperatures without humidity control, and shoots of 3-week-old plants were harvested. Each sample was a pool of five plant shoots, and samples were harvested from six independent experiments. For quantification of *AT1G19410* (*ANH*) mRNA levels, Col-0 and *ddcc*^88^ plants were grown for 10 days as described above, then a 12-h cold treatment (4 °C) was applied to induce and detect the expression of *ANH*^121^. *ANH* transcript abundance was analysed from five independent experiments with 25 plant shoots pooled per experiment. Total RNA was extracted using the SV Total RNA Isolation System (Promega, cat. no. Z3101). One microgram of total RNA was used for first-strand cDNA synthesis using SuperScript IV Reverse Transcriptase (Invitrogen, 18090050) and Oligo(dT)15 Primer (Invitrogen, 18418012) in a final volume of 25 μl, according to the manufacturer’s instructions. For qRT–PCR, 25 ng of first-strand cDNA was used as template. qRT–PCR was performed in triplicate using the CFX Connect Real Time PCR Detection System (Bio-Rad). cDNA amplification was monitored using SensiFAST SYBR No-ROX One-Step Kit (Biolone, Bio-72005) at an annealing temperature of 60 °C. *UBQ10* (AT4G05320) was used as an internal control. The primer sequences used for the analysis of *PROT1*, *ANH* and *UBQ10* are listed in Supplementary Table 17. Relative transcript levels (RTL) of genes of interest (GOI) compared with *UBQ10* were determined using the equation RTL = [(*E*)^−Ct^]^GOI^/[(*E*)^−Ct^]^UBQ10^.

### Analysis of methylation upstream of FLC

Methylation was analysed upstream of *FLC* in reference to previously described regions ‘X’ and ‘Y’^87^ (Supplementary Fig. 11a). The borders of region X were set as 3,180,248-3,180,730 and the borders of region Y as 3,181,100-3,181,451. Region X was split into two separate regions (X1: 3,180,248-3,180,350 and X2: 3,180,351-3,180,730), as methylation of these regions showed different patterns of variation within the population (Supplementary Fig. 11a–d). Methylation levels in each region were calculated per accession. Only accessions with mean coverage over a given region of at least five reads per CG site and three reads each per CHG and CHH site were included for subsequent analysis. We identified 18 accessions methylated at X2 in all three contexts (>30% mCG, >5% mCHG and >1% mCHH; *Arabidopsis* accession IDs: 6092, 6102, 6111, 6136, 6137, 6145, 6150, 6907, 7430, 8247, 9524, 9703, 9759, 9777, 9790, 9839, 9850 and 9900). Of these, 13 belonged to haplogroups with multiple accessions (Supplementary Table 16). Flowering times and expression of *FLC* were available for 9 of these accessions (Supplementary Figs. 12e and 13e).

### Quantification of minerals in plant samples

WT and mutant plants were grown in four biological replicates to analyse the accumulation of minerals in the shoots. Oven-dried samples (~15 mg) were placed in a vessel (Environmental Express, cat. no. SC415) with 1 ml of nitric acid 65% (EMD Millipore cat. no. 1.00456.2500) and hydrogen peroxide 30% (Sigma-Aldrich cat. no. H3410-1L) and left at room temperature overnight. The samples were then digested using an Environmental Express Hotblock digestion system (cat. no. SC196) set at 80 °C for 8 h. Microwave-induced plasma optical emission spectrometer 4210 (MP-AES Agilent Technologies) coupled with an autosampler SPS4 (Agilent Technologies) was used to quantify K, Mg, Mn and Zn at 769.897 nm, 280.271 nm, 403.076 nm and 202.548 nm, respectively. Standard curves for each element were used to determine mineral concentrations in samples.

### Plant materials, growth conditions and phenotyping

For relative fitness phenotyping under drought and heat stress, seeds of *Arabidopsis* Col-0 accessions and homozygous T-DNA insertion mutant lines for *PROT1* (*prot1*-*1*; SALK_030711C and *prot1*-*2*; SALK_018050C) and *ANH* (*anh*-*1*; SALK_098287C and *anh*-*2*; SALK_036488C) were obtained from Nottingham Arabidopsis Stock Centre. Seeds were stratified at 4 °C for 7 days and germinated in 9-cm pots containing vermiculite. Each pot contained four plants. Plants were grown in a chamber with cycles of 16 h light (120 µE m^−2^ s^−1^) and 8 h dark, with 16 °C night and 27 °C day temperatures to induce heat stress. For well-watered conditions soil water content (SWC) was maintained at 60%, and 25% SWC was used for drought stress. Each pot was weighed daily to adjust SWC. Survival to fruit for Col-0 WT plants and *prot1* and *anh* mutant plants was scored before harvesting under heat or joint heat and drought stress. The number of seeds produced by surviving plants was recorded as a measure of fecundity. The fitness of each genotype under heat or combined heat and drought stress was calculated as a product of per cent survival and average fecundity during each experiment. The relative fitness of *prot1* and *anh* was estimated with respect to the average fitness of Col-0 within each condition. To understand the phenotypes that could contribute to differences in relative fitness of *prot1* and *anh* mutant plants, the three genotypes were phenotyped for shoot biomass and fertility. Shoot biomass for Col-0, *prot1* and *anh* plants was measured as shoot dry weight at maturity. Fertility was scored as a percentage of flowers producing siliques.

For flowering time phenotyping, seeds of *Arabidopsis* Col-0 accessions and homozygous T-DNA insertion mutant lines *AT1G51820* (*at1g51820-*1; SALK_208927 and *at1g51820-2*; SALK_055952), *AT1G18210* (*at1g18210-1*; GABI_826B09 and *at1g18210-2*; SALK_075633), *AT3G43860* (*at3g43860-1*; SALK_201540 and *at3g43860-1*; GABI_129G07), *AT3G09530* (*at3g09530-1*; SALK_034560 and *at3g09530-2*; SALK_023893), *AT1G26795* (*at1g26795-1*; SALK_124311 and *at1g26795-1*; SALK_124319) and *AT4G33560* (*at4g33560-1*; SALK_133653) were obtained from Nottingham Arabidopsis Stock Centre. T-DNA insertion mutant lines (*AT1G09725* (*at1g09725*; CS821762), *AT4G18370* (*at4g18370-1*; SALK_099162C and *at4g18370-2*; SALK_036606C), *AT4G02550* (*at4g02550-1*; SALK_136283C and *at4g02550-2*; SALK_028806C), *AT1G70920* (*at1g70920*; CS863888), *AT5G61850* (*lfy-1* and *lfy-9*), *AT2G16200* (*at2g16200*; SALK_082813), *AT1G50470* (*at1g50470*; SALK_200371C), *AT2G13570* (*at2g13570*; SALK_085886C), *AT4G22910* (*at4g22910-1*; SALK_083656C and *at4g22910-2*; SALK_101689C), *AT1G28650* (*at1g28650*; SALK_010911C); *AT2G40815* (*at2g40815-1*; SAIL_138_E02 and *at2g40815-2*; SALK_023214C) and *AT1G28135* (*at1g28135*; SALK_017094)) for mineral content analysis were obtained from Arabidopsis Biological Resource Center. Seeds were stratified at 4 °C for 7 days and germinated in 9-cm pots containing vermiculite, with each pot containing three plants. Plants were grown in a chamber with cycles of 16 h light (120 µE m^−2^ s^−1^) and 8 h dark, with 16 °C constant temperature. The flowering time of each genotype was scored as the number of days to the appearance of the first flower.

### Reporting summary

Further information on research design is available in the Nature Portfolio Reporting Summary linked to this article.

## Supplementary information

Supplementary Information Supplementary Figs. 1–13.

Reporting Summary

Supplementary Tables Supplementary Tables 1–23.

## Data Availability

Newly generated RNA-seq and bisulfite sequencing data from plants with mosaic gbM are available at GEO under accession number GSE183785. In addition, previously published datasets were used as follows: GSE43857: 1001 genomes project bisulfite sequencing data^40^; GSE80744: 1001 genomes project RNA-seq data^40^; PRJEB54036: RNA-seq *met1* mutant data from sixteen *Arabidopsis* accessions^75^; GSE122394: RNA-seq *met1* mutant data from Col-0 leaf and seedling^54^; and GSE93584: RNA-seq *met1* mutant data from Col-0 inflorescence^77^.
