# Supplementary material for: Gene body methylation regulates gene expression and mediates phenotypic diversity in natural Arabidopsis populations
Source: Nat Plants. 2025 Sep 12;11(10):2084–99. doi: 10.1038/s41477-025-02108-4 (PMC12537493; doi:10.1038/s41477-025-02108-4)
Supplement: Supplementary file 1 — Supplementary Figs. 1–13. [file 41477_2025_2108_MOESM1_ESM.pdf]

nature plants

Supplementary information https://doi.org/10.1038/s41477-025-02108-4

Gene body methylation regulates gene expression and mediates phenotypic diversity in natural *Arabidopsis* populations

In the format provided by the authors and unedited

Contents:

Supplementary Figures 1-13

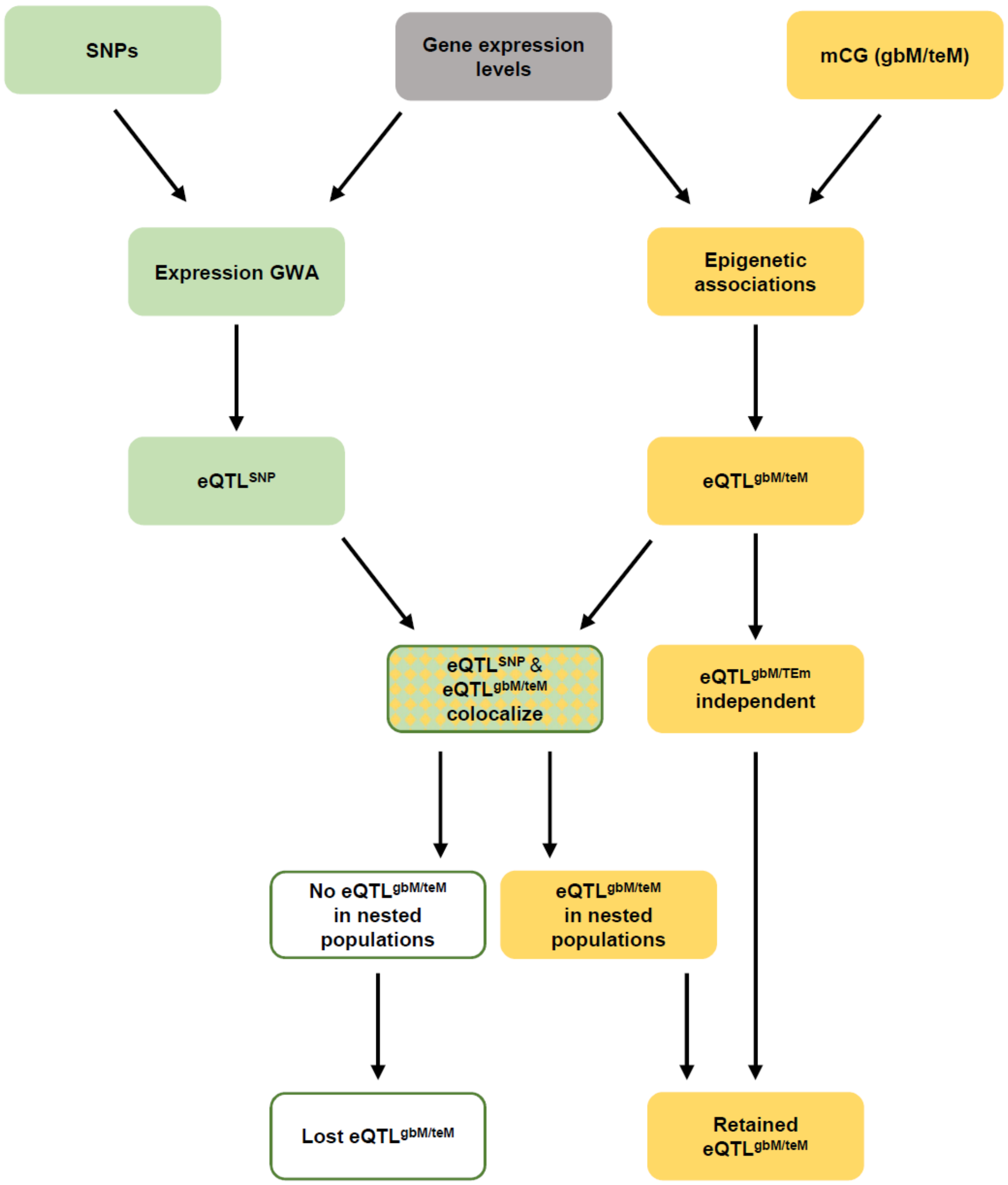

Supplementary Fig. 1. Schematic of the pipeline used to examine the effects of genetic variation on eQTL$^{gbM/teM}$ gene expression. GWA analyses were performed using SNP data to identify genetic *cis* eQTLs (eQTL^{SNP}) underlying expression variance of eQTL$^{gbM/teM}$ genes. If a eQTL$^{gbM/teM}$ colocalized with a eQTL^{SNP}, two nested populations were generated based on the GWA SNP. Association of intragenic DNA methylation with gene expression was re-examined within nested populations. eQTL$^{gbM/teM}$ were retained if they did not colocalize with eQTLs^{SNP} or if a eQTL$^{gbM/teM}$ was detected in at least one nested population.

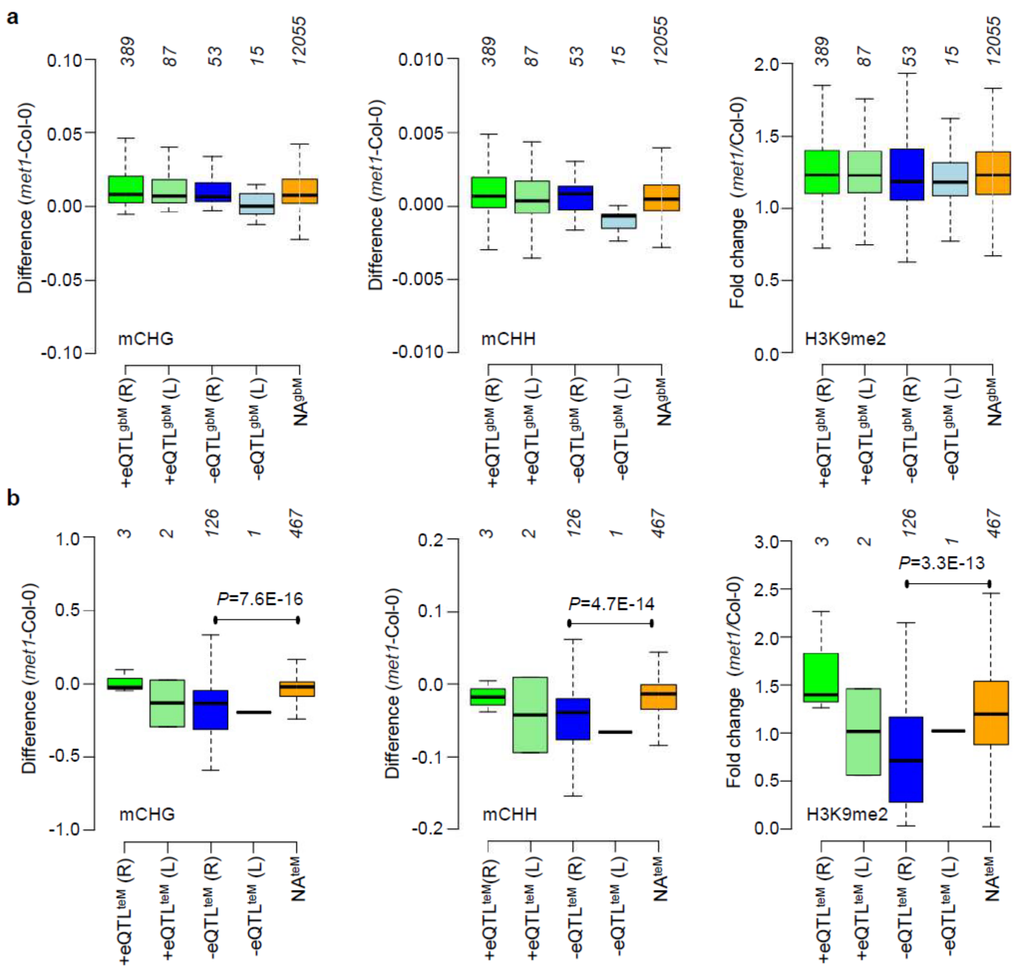

Supplementary Fig. 2. Changes in non-CG methylation and H3K9me2 in *met1*. (**a** and **b**) CHG and CHH methylation and H3K9me2 (dimethylation of lysine 9 of histone H3) in *met1* compared to Col-0 of Bonferroni (α=0.05) eQTL^{gbM} (**a**) and eQTL^{teM} (**b**) genes. (R) and (L) denote eQTL genes retained (R) or lost (L) after accounting for genetic variation, whereas NA indicates non-associated genes. Published data[78] for non-CG methylation and H3K9me2 were analyzed for genes with intragenic DNA methylation (gbM; teM) in Col-0. Sample medians are shown by the center lines; box edges represent the 25th and 75th percentiles. Whiskers extend to 1.5 times the interquartile range. Numbers of genes within each group are indicated. *P*, two-tailed Student's t-test; the remaining comparisons with NA genes are not significant.

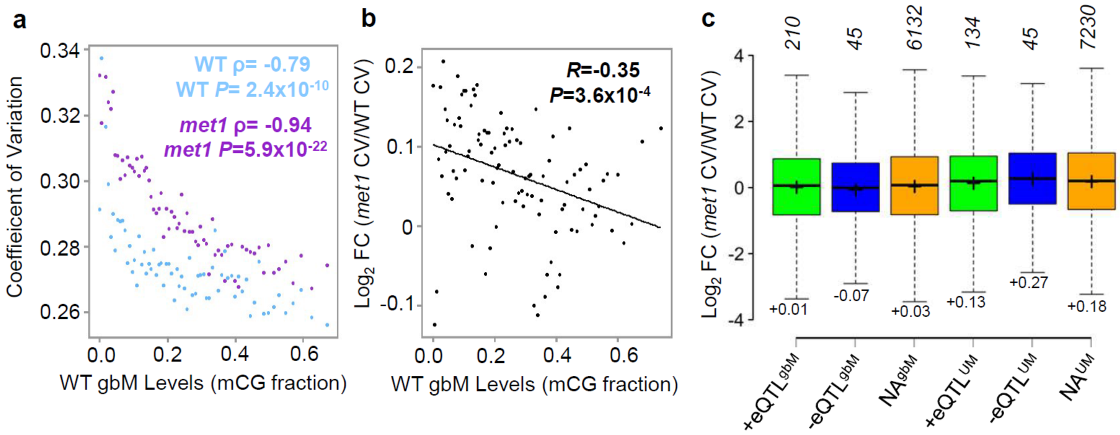

Supplementary Fig. 3. Gene expression variability in wild type and *met1*. (**a**) Relationship between the gene expression coefficient of variation (CV) across biological replicates in WT (blue) and *met1* (purple) and WT gbM level across 16 accessions. Only genes with unchanged expression in *met1* ($p > 0.05$, $\log_2$ fold change between -1 and 1) were used (n=12,616). Genes were grouped by gbM levels. ρ and P values correspond to Spearman's rank correlation coefficient. (**b**) Relationship between the $\log_2$ fold CV change in *met1* compared to WT and the gbM level across 16 accessions. Only genes with unchanged expression in *met1* were used. (**c**) Log_2 fold CV change in *met1* compared to WT of Bonferroni (α=0.05) retained eQTL^{gbM} genes that are methylated in a given accession (eQTL^{gbM}) and eQTL^{gbM} genes that are unmethylated in a given accession (eQTL^{UM}) across 16 accessions. Sample medians and means are indicated with center lines and crosses, respectively, means are noted below the plots, and box edges represent the 25th and 75th percentiles. Whiskers extend to 1.5 times the interquartile range. Numbers of unique genes within each group are noted above the plots. NA indicates non-associated genes.

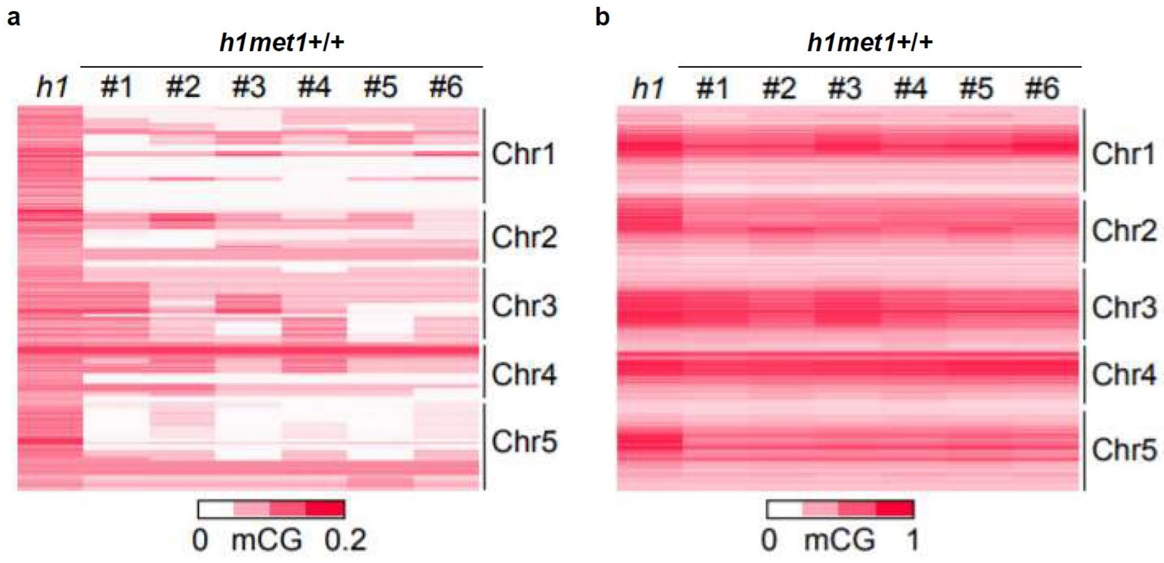

Supplementary Fig. 4. DNA methylation in *h1* and *h1met1*+/+ plants. (**a** and **b**) Heat maps showing CG methylation in gene bodies (**a**) and TEs (**b**) in *h1* or *h1met1*+/+ leaves. Six independent *h1met1*+/+ plants were isolated from segregating *h1met1*+/- plants.

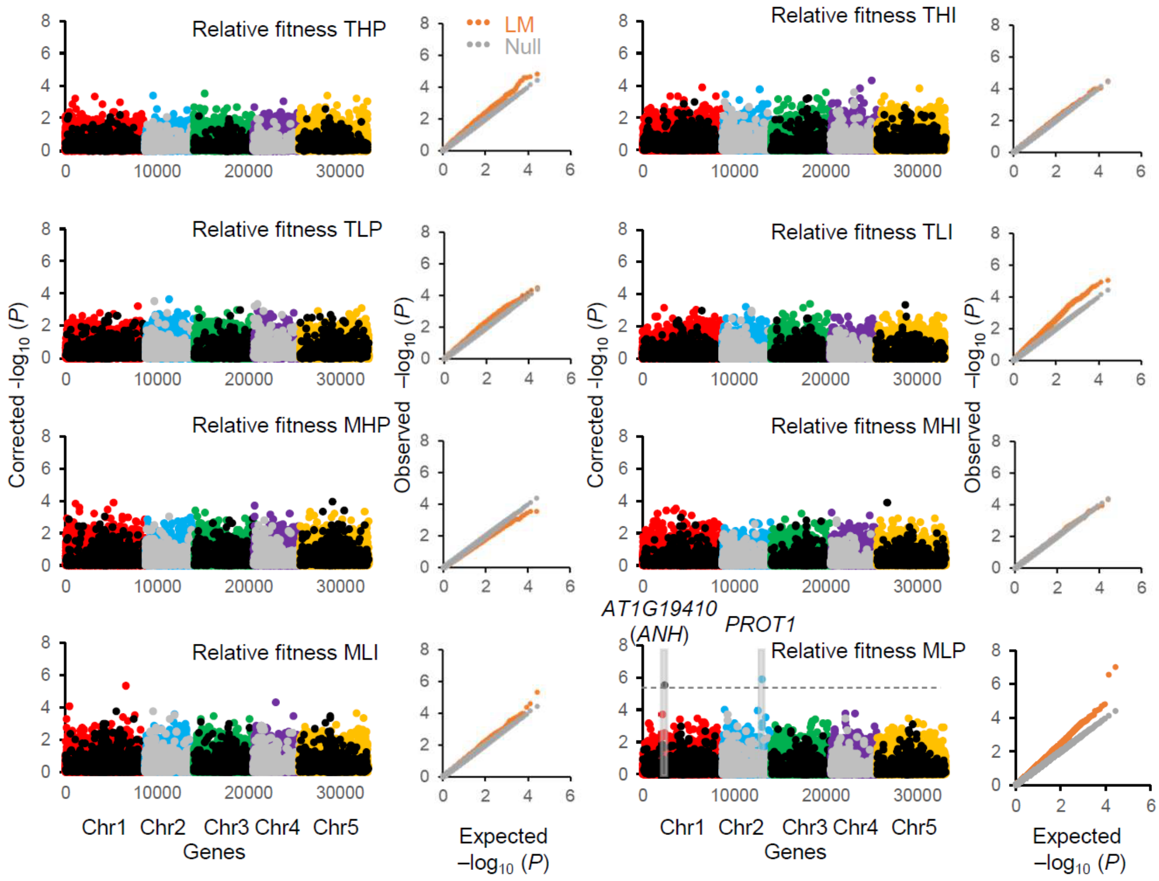

Supplementary Fig. 5. Associations between intragenic methylation and relative fitness. Manhattan and QQ plots for relative fitness epiGWA in THP, THI, TLP, THI, MHP, MHI, MLP, and MLI conditions (T, Tübingen; M, Madrid; H, high rainfall; L, low rainfall; P, high plant density; and I, individual plants). QQ plots compare the distribution of observed (orange dots) and expected (diagonal grey dots) -$\log_{10} P$ values. To account for confounding effects of population stratification, genomic control factor λ was used to correct association statistics. Corrected -$\log_{10} P$ values for gbM (colored dots) and teM (grey and black dots) markers are plotted in Manhattan plots. Horizontal dashed line shows 0.05 FDR.

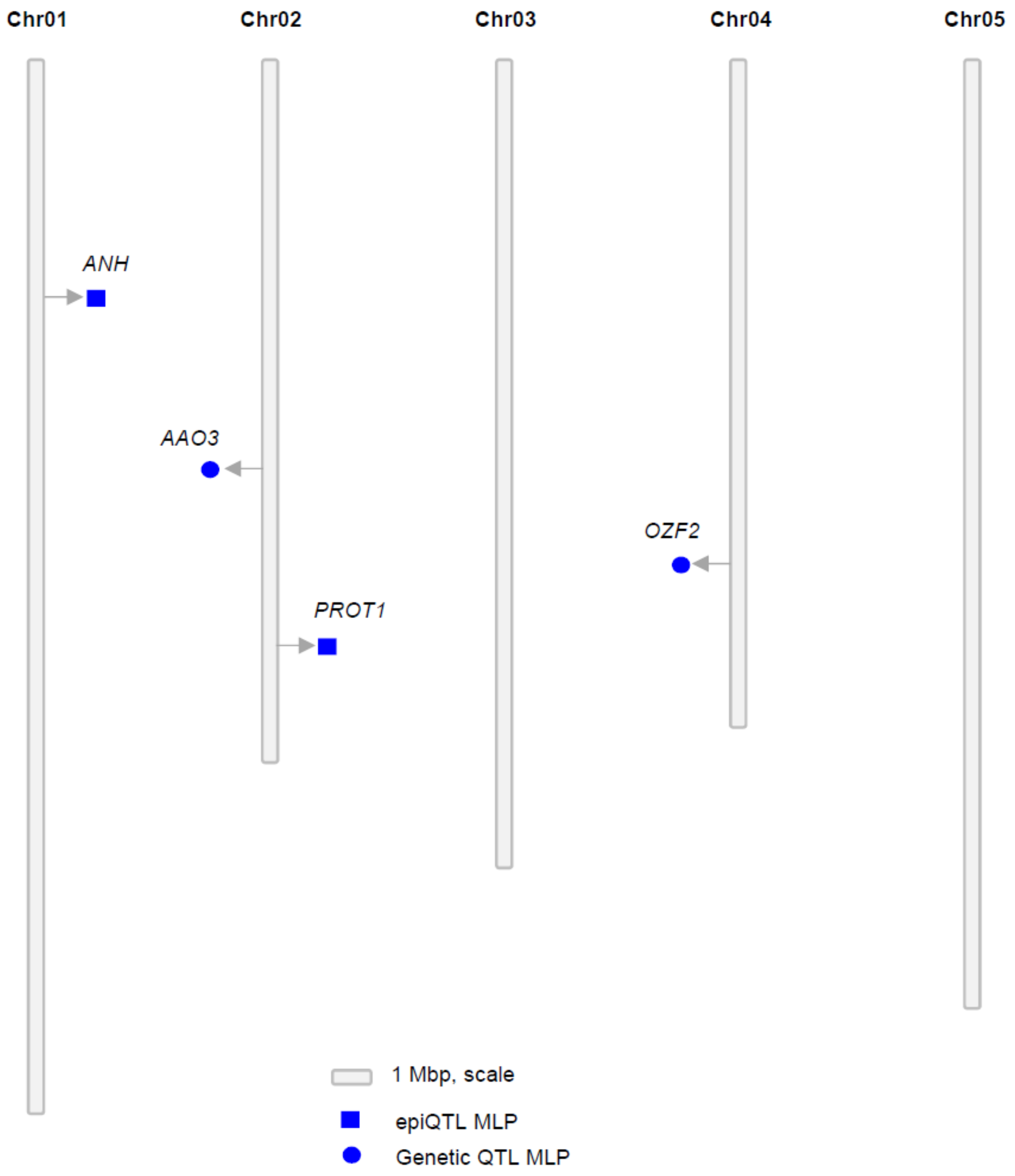

Supplementary Fig. 6. Distinct epigenetic and genetic QTLs are associated with relative fitness under MLP conditions. *Arabidopsis* physical map showing the positions of epigenetic and genetic QTLs associated with relative fitness under MLP conditions. Five chromosomes of *Arabidopsis* are presented in Mb scale. Positions of epigenetic QTLs are shown with squares to the right of chromosomes, and circles on the left depict positions of genetic QTLs.

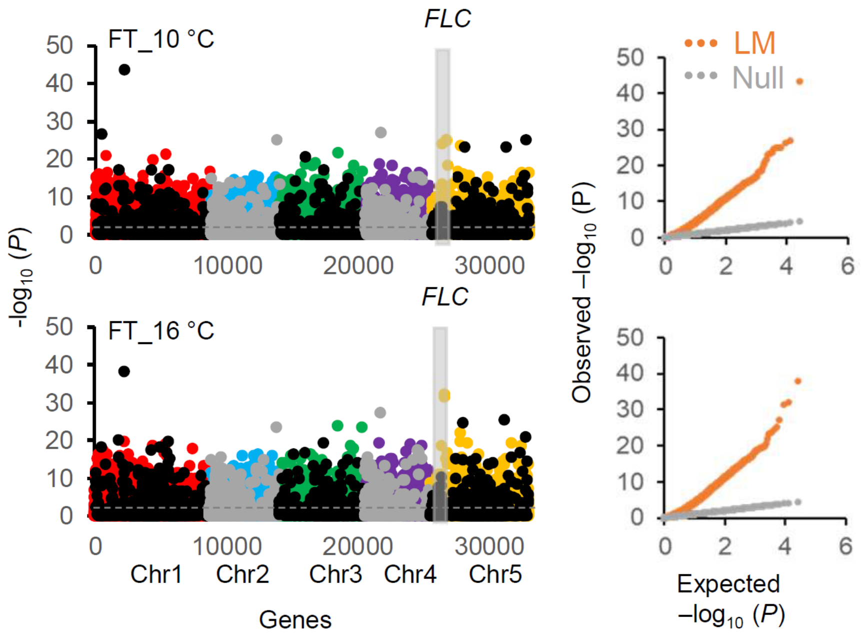

Supplementary Fig. 7. Population structure confounds association statistics of linear model epiGWA mapping for flowering phenotypes. Manhattan and QQ plots of epiGWA mapping for flowering phenotypes (FT_10°C and FT_16°C). Associations between intragenic DNA methylation (mCG) levels and flowering time were examined using a linear model. QQ plots compare the distribution of observed (orange dots) and expected (diagonal grey dots) -$\log_{10} P$ values. Colored dots in Manhattan plots represent gbM markers and grey and black dots correspond to teM markers. Horizontal dashed line shows 0.05 FDR. mCG levels of around 7,500 genes are significantly associated with flowering phenotypes. We expect some of these associations to be real, for example the well-known flowering regulator, *FLC*. However, it is difficult to distinguish true associations from spurious ones. Strong inflation of observed *P* values compared to expectation also results in high values for genomic control factor (λ) (Table S10). Genomic control method in cases of high λ can be anticonservative, thus is not recommended to account for population stratification[114,115]. Linear model GWA mapping with genomic control is generally considered inappropriate for structured phenotypes[112] and is not used in this study.

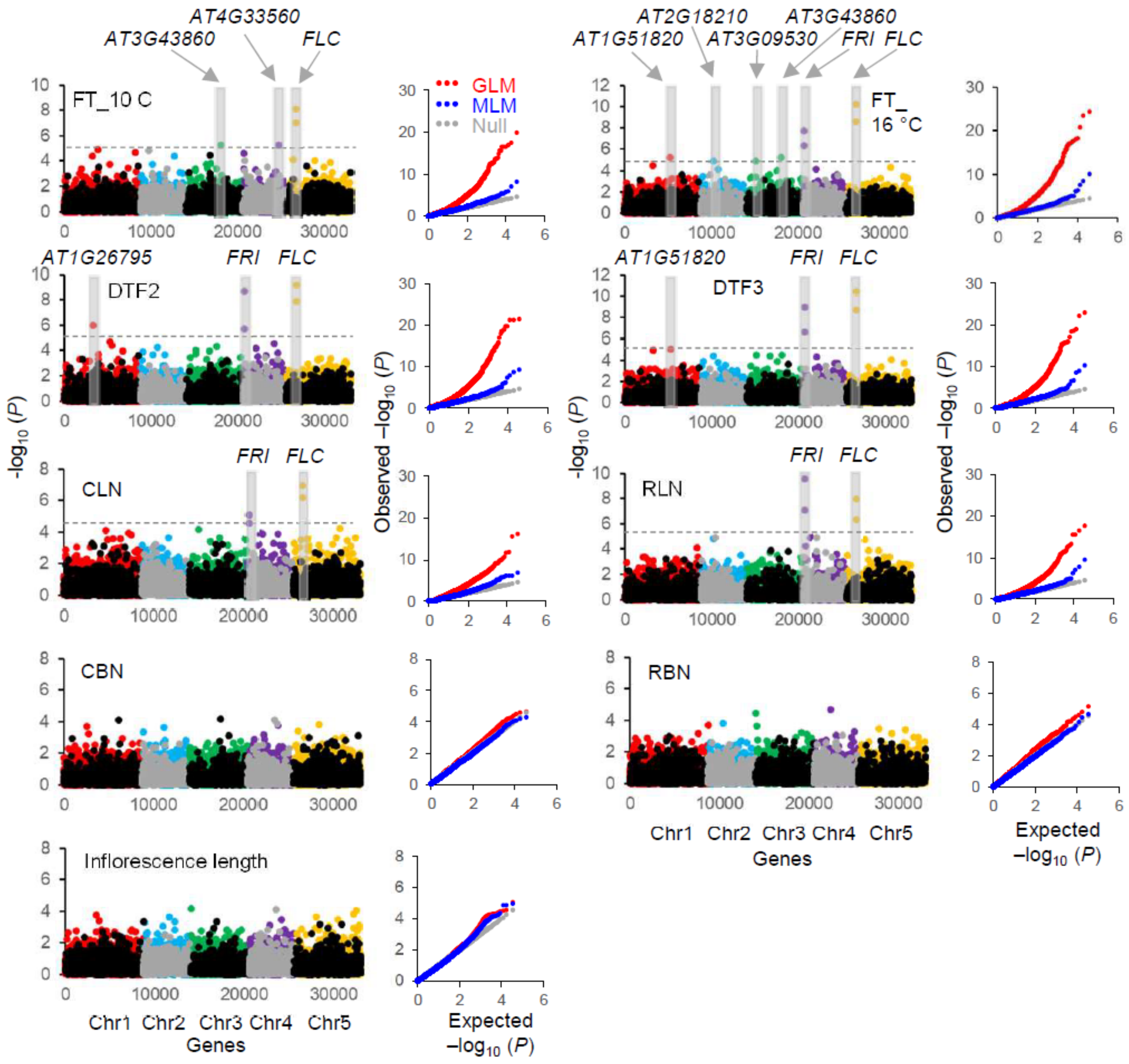

Supplementary Fig. 8. Mixed linear model corrects population structure in epiGWA analyses for flowering phenotypes. EpiGWA mapping for nine flowering related phenotypes (flowering time at 10°C (FT_10°C), flowering time at 16°C (FT_16°C), number of days for inflorescence stalk to reach 1 cm (DTF2), number of days to opening of the first flower (DTF3), number of cauline leaves (CLN), number of rosette leaves (RLN), cauline branch number (CBN), primary number of inflorescence branches (RBN), and length of primary inflorescence stalk. Associations between epiallelic states (UM and gbM; UM and teM) of genes and each flowering phenotype were examined using a generalized linear model (GLM) or a mixed linear model (MLM). QQ plots compare the distribution of observed GLM epiGWA (red dots), observed MLM epiGWA (blue dots) and expected (diagonal grey dots) -$\log_{10}$ *P* values. In Manhattan plots, colored dots depict gbM markers and teM markers are shown using grey and black dots. Horizontal dashed lines show MLM 0.05 FDR. Epigenetic flowering QTLs (passing 0.05 FDR) are highlighted with grey bars.

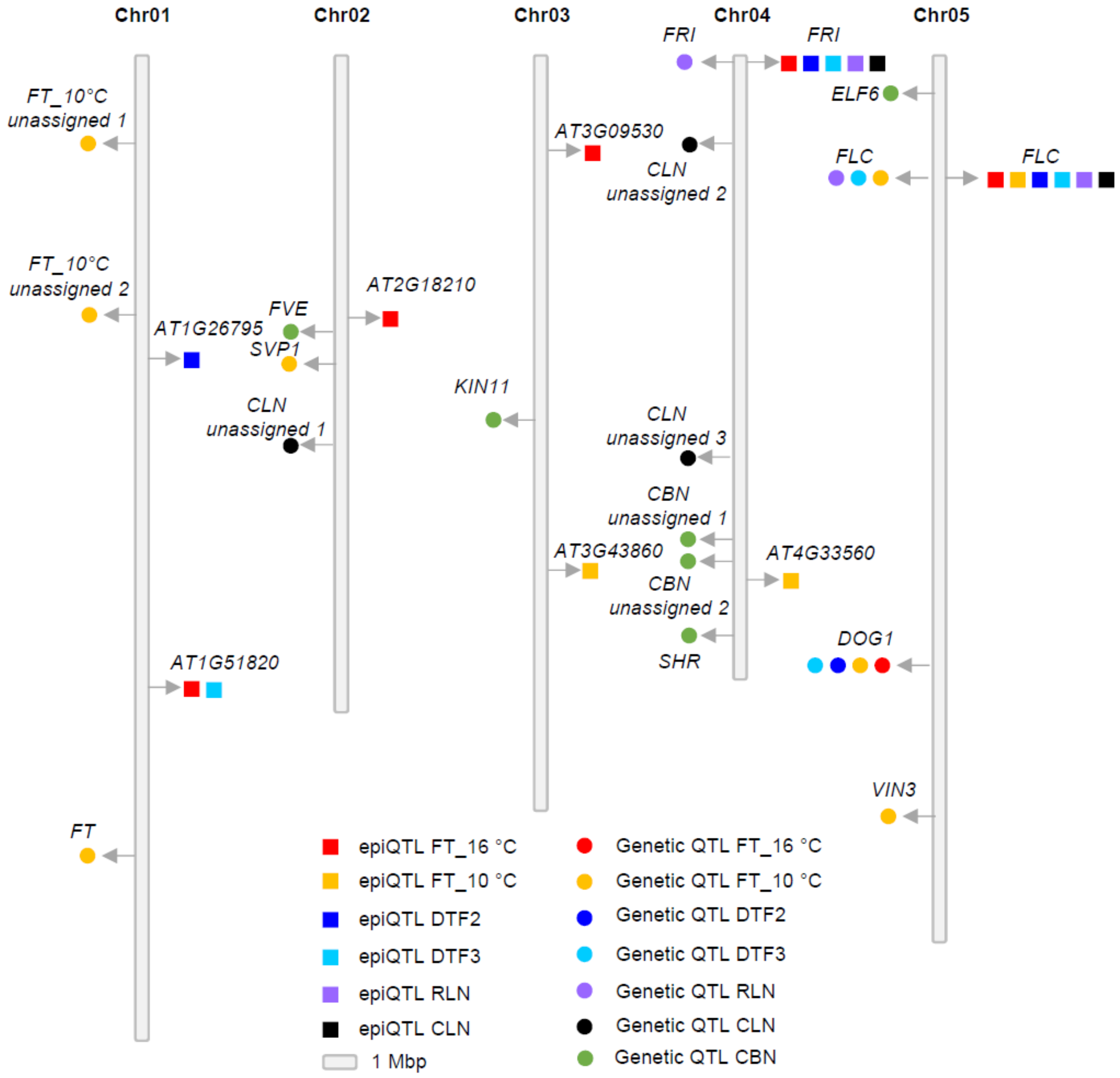

Supplementary Fig. 9. Epigenetic and genetic QTLs associated with flowering phenotypes are largely independent. *Arabidopsis* physical map showing the positions of epigenetic and genetic QTLs associated with flowering phenotypes. Five chromosomes are shown in Mb scale. Positions of epigenetic QTLs are shown with squares to the right of chromosomes, and circles on the left depict positions of genetic QTLs.

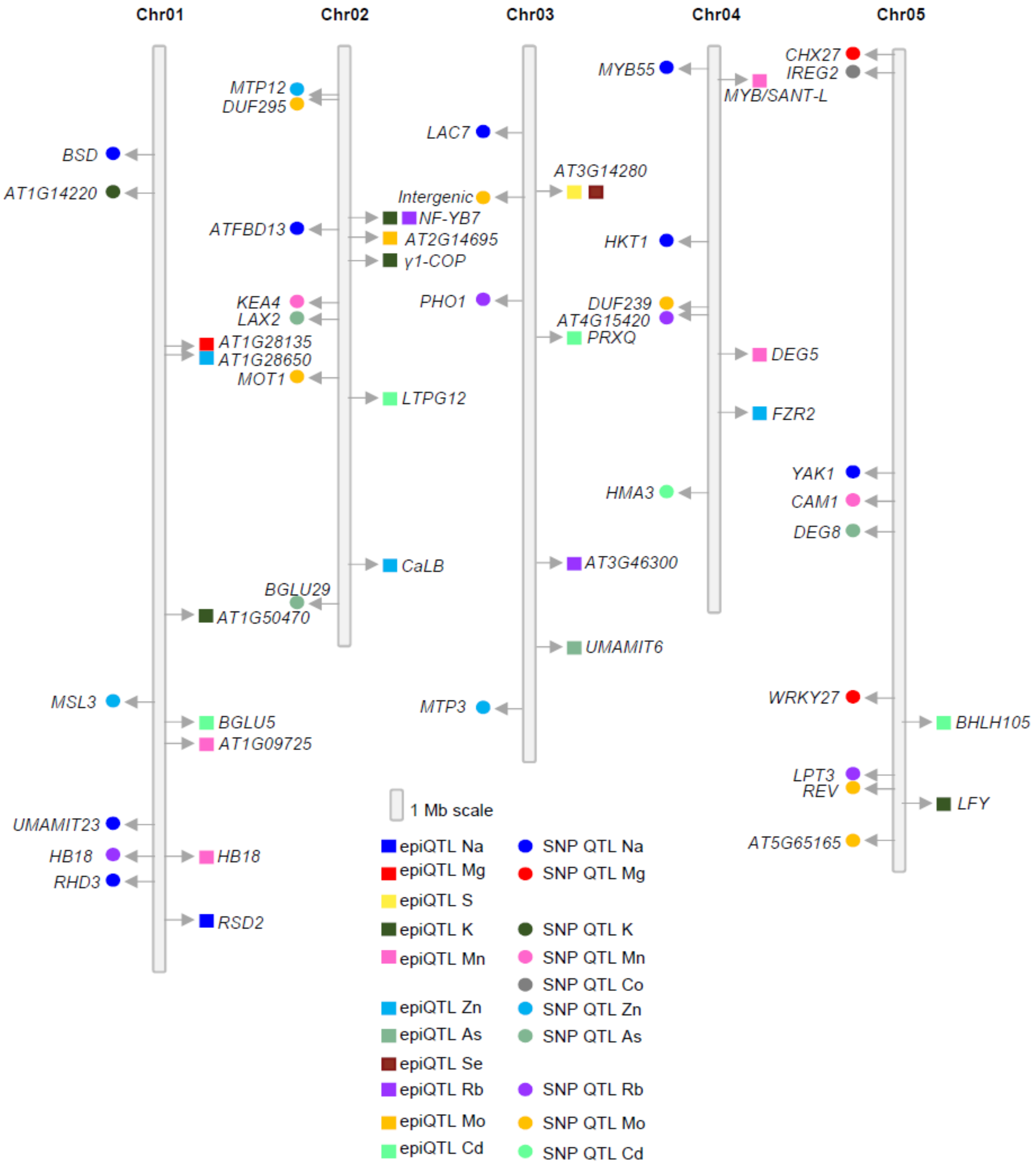

Supplementary Fig. 10. Epigenetic and genetic QTLs associated with Arabidopsis leaf mineral accumulation are largely independent. *Arabidopsis* physical map showing the positions of epigenetic and genetic QTLs associated with the levels of sodium (Na), magnesium (Mg), sulfur (S), potassium (K), manganese (Mn), cobalt (Co), zinc (Zn), arsenic (As), selenium (Se), rubidium (Rb), molybdenum (Mo), and cadmium (Cd) phenotypes. Five chromosomes are shown in Mb scale. Positions of epigenetic QTLs are shown with squares to the right of chromosomes, and circles on the left depict positions of genetic QTLs.

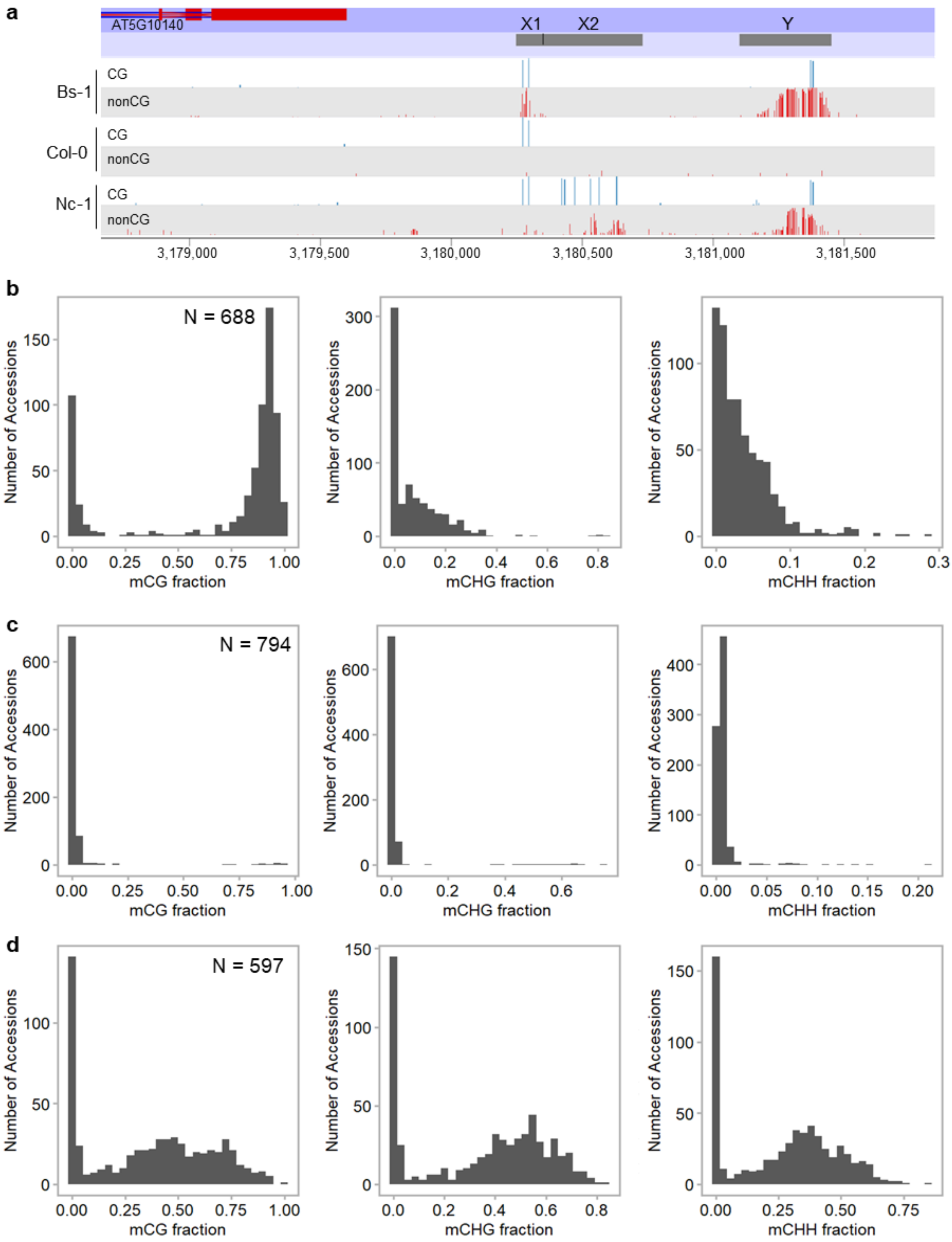

Supplementary Fig. 11. DNA methylation upstream of *FLC*. (**a**) Genome browser view of previously described[87] methylated regions upstream of *FLC* (AT5G10140), with mCG (blue) and non-CG methylation (red) shown for three example accessions illustrating distinct methylation patterns. We subdivided region X into sub-regions X1 and X2, as methylation of these regions showed different patterns of variation within the population. (**b-d**) Methylation levels of X1 (**b**), X2 (**c**) and Y (**d**) across *Arabidopsis* accessions. Only accessions with mean coverage over the given region of at least 5 reads per CG site and 3 reads per CHG and CHH site, respectively, were included. N indicates the number of accessions analyzed for each region. Note that methylation of X1 is common in the population (especially mCG), and methylation in all contexts is common at Y. In contrast, methylation of X2 is rare, with just 18 accessions showing substantial methylation (Methods). Methylation of X2 has been linked[87] to lower *FLC* expression and earlier flowering.

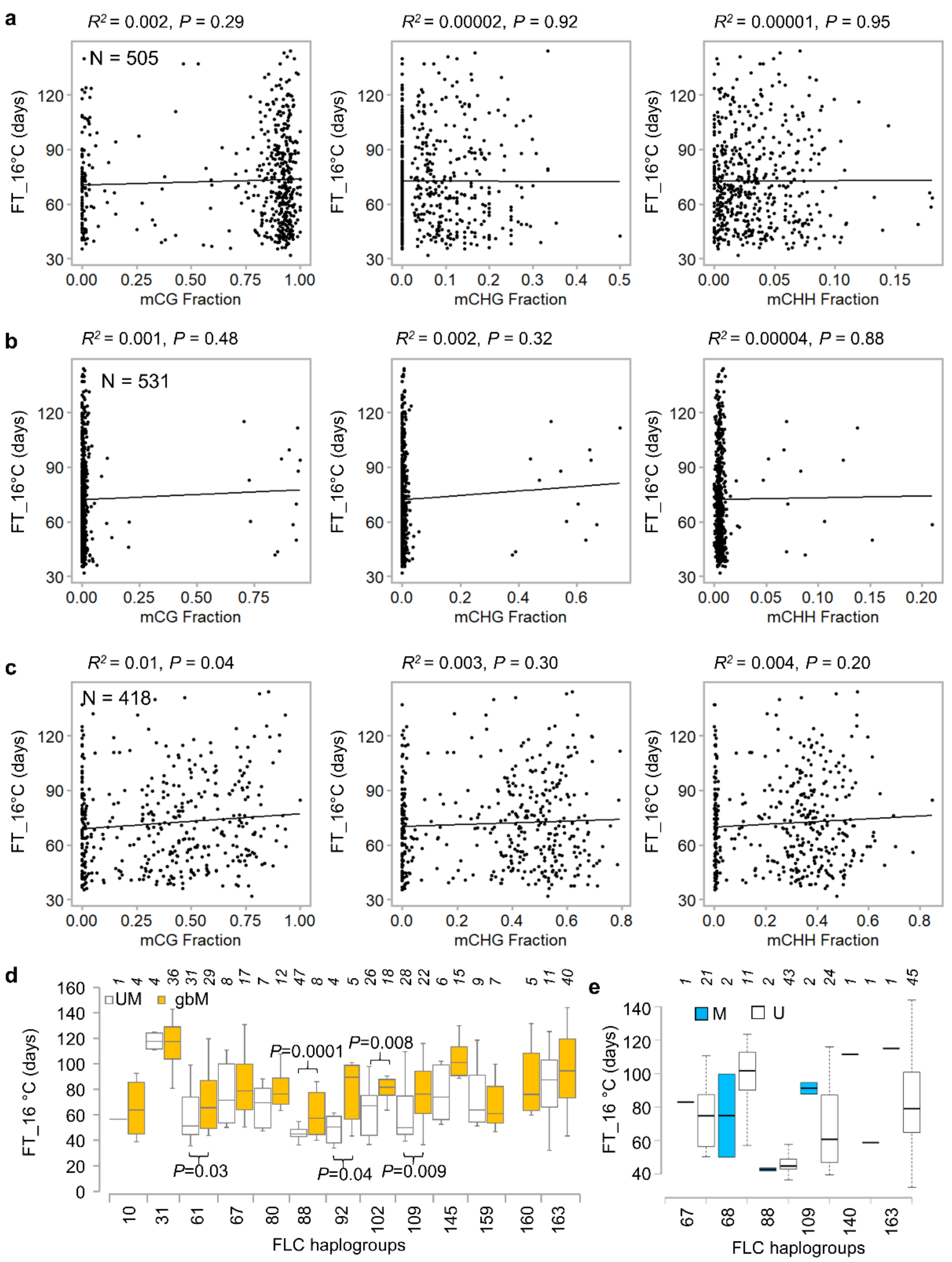

Supplementary Fig. 12. Association between methylation upstream of *FLC* and flowering time. (**a-c**) Correlation of flowering time (FT_16°C) with methylation in the CG, CHG and CHH contexts in regions X1 (**a**), X2 (**b**) and Y (**c**) upstream of *FLC*, as illustrated in Supplementary Fig. 11a. Only accessions with flowering time and *FLC* expression data, as well as mean coverage over a given region of at least 5 reads per CG site and 3 reads per CHG and CHH site, respectively, were included. N indicates the number of included accessions for a given region. (**d**) Association of *FLC* epiallelic states with FT_16°C in thirteen *FLC* haplogroups, as in Fig. 4a, excluding accessions with methylation at X2. (**e**) Flowering time of accessions with methylated X2 (M, blue) and unmethylated X2 (U, white) within haplogroups where M and U accessions are present. None of the comparisons between M and U accessions are significant, as expected from the small number of M accessions. However, note that although methylation at X2 has been proposed to accelerate flowering[87], in four of the six haplogroups M accessions flower later than U accessions. Center lines within boxplots (**d**,**e**) correspond to medians. The boundaries of the box are the first and third quartiles and whiskers extend to 1.5-fold the interquartile range. *P* values correspond to two-tailed Student's t-test. Numbers of accessions involved are indicated above the plots.

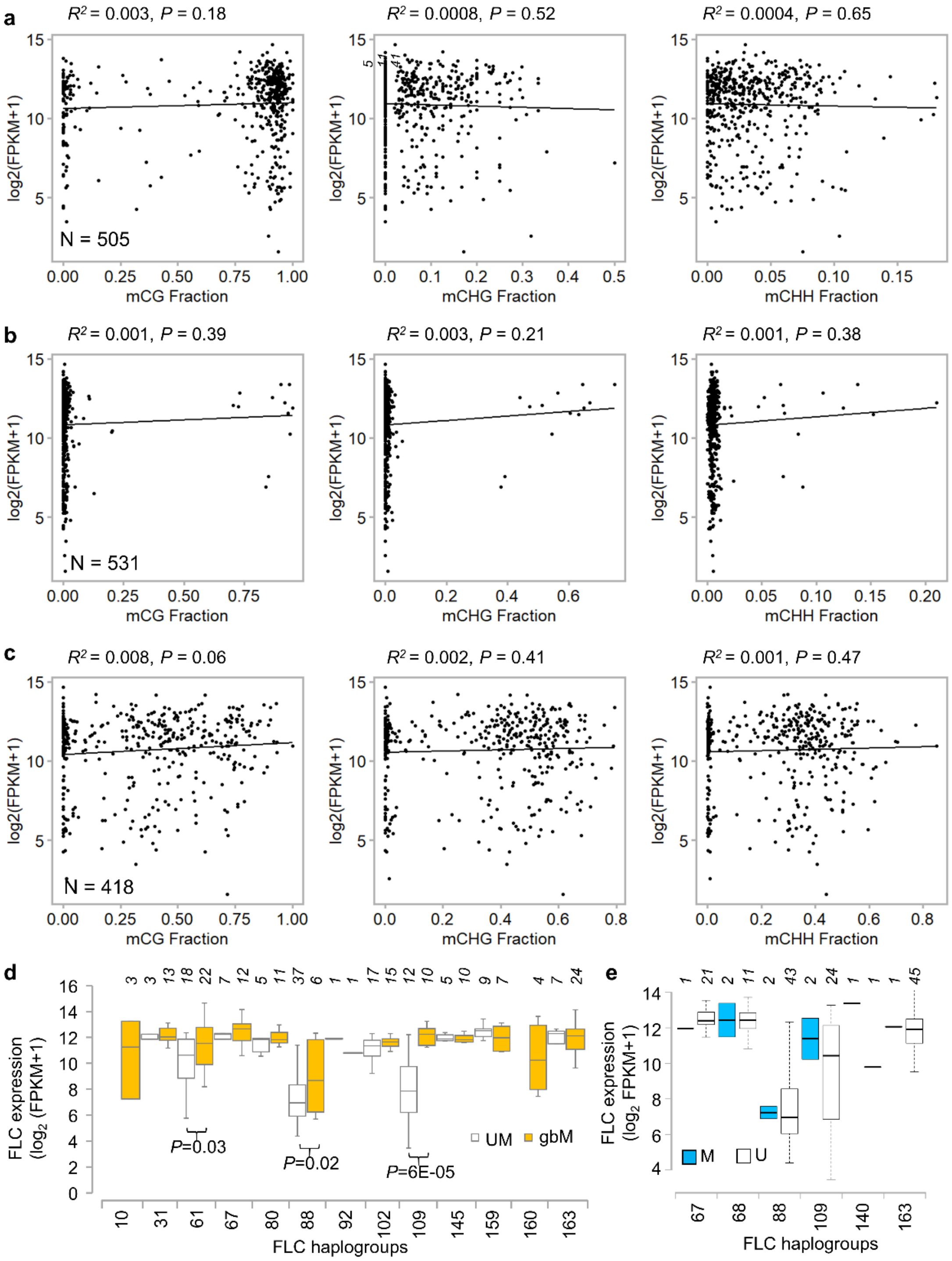

Supplementary Fig. 13. Association between methylation upstream of *FLC* and *FLC* expression. (**a-c**) Correlation of *FLC* expression with methylation in the CG, CHG and CHH contexts in regions X1 (**a**), X2 (**b**) and Y (**c**) upstream of *FLC*, as illustrated in Supplementary Fig. 11a. Only accessions with flowering time and *FLC* expression data, as well as mean coverage over a given region of at least 5 reads per CG site and 3 reads per CHG and CHH site, respectively, were included. N indicates the number of included accessions for a given region. (**d**) Association of *FLC* epiallelic states with *FLC* expression in thirteen *FLC* haplogroups, as in Fig. 4b, excluding accessions with methylation at X2. (**e**) *FLC* expression of accessions with methylated X2 (M, blue) and unmethylated X2 (U, white) within haplogroups where M and U accessions are present. None of the comparisons between M and U accessions are significant, as expected from the small number of M accessions. However, note that although methylation at X2 has been proposed to reduce *FLC* expression[87], in four of the six haplogroups M accessions have higher *FLC* expression than U accessions. Boxplots are as in Supplementary Fig. 12d,e. *P* values correspond to two-tailed Student's t-test.
