# Supplementary material for: Gene body methylation regulates gene expression and mediates phenotypic diversity in natural Arabidopsis populations
Source: Nat Plants. 2025 Sep 12;11(10):2084–99. doi: 10.1038/s41477-025-02108-4 (PMC12537493; doi:10.1038/s41477-025-02108-4)
Supplement: Supplementary file 2 — Reporting Summary [file 41477_2025_2108_MOESM2_ESM.pdf]

nature portfolio

Corresponding author(s): Daniel Zilberman

Last updated by author(s): Aug 5, 2025

# Reporting Summary

Nature Portfolio wishes to improve the reproducibility of the work that we publish. This form provides structure for consistency and transparency in reporting. For further information on Nature Portfolio policies, see our Editorial Policies and the Editorial Policy Checklist.

## Statistics

For all statistical analyses, confirm that the following items are present in the figure legend, table legend, main text, or Methods section.

| n/a | Confirmed | |
|---|---|---|
| ☐ | ☒ | The exact sample size (*n*) for each experimental group/condition, given as a discrete number and unit of measurement |
| ☐ | ☒ | A statement on whether measurements were taken from distinct samples or whether the same sample was measured repeatedly |
| ☐ | ☒ | The statistical test(s) used AND whether they are one- or two-sided<br>*Only common tests should be described solely by name; describe more complex techniques in the Methods section.* |
| ☐ | ☒ | A description of all covariates tested |
| ☐ | ☒ | A description of any assumptions or corrections, such as tests of normality and adjustment for multiple comparisons |
| ☐ | ☒ | A full description of the statistical parameters including central tendency (e.g. means) or other basic estimates (e.g. regression coefficient) AND variation (e.g. standard deviation) or associated estimates of uncertainty (e.g. confidence intervals) |
| ☐ | ☒ | For null hypothesis testing, the test statistic (e.g. *F*, *t*, *r*) with confidence intervals, effect sizes, degrees of freedom and *P* value noted<br>*Give P values as exact values whenever suitable.* |
| ☐ | ☒ | For Bayesian analysis, information on the choice of priors and Markov chain Monte Carlo settings |
| ☐ | ☒ | For hierarchical and complex designs, identification of the appropriate level for tests and full reporting of outcomes |
| ☐ | ☒ | Estimates of effect sizes (e.g. Cohen's *d*, Pearson's *r*), indicating how they were calculated |

*Our web collection on statistics for biologists contains articles on many of the points above.*

## Software and code

Policy information about availability of computer code

| | |
|---|---|
| Data collection | No software was used in data collection. |
| Data analysis | Analysis of methylation data: BSMAP (v2.90)<br>Analysis of RNA-seq data: HiSat2 (v2.11.2), samtools (v1.18),<br>Further analysis of bisulfite and RNA-seq data, statistical analyses and creation of plots: R, including additional packages ggplot2 (v3.4.1), Rsubread (v2.12.3), DESeq2 (v1.38.3), dplyr (v1.1.1), GenomicRanges (v1.50.2), data.table (v1.14.8), matrixStats (v0.63.0), scales (v1.2.1), tidyr (v1.3.0)<br>Fitting of a regression model - Trejo Banos et al., 2020<br>Correlation analysis: SigmaPlot 1.0<br>epiGWAS: MLM and GLM implemented in TASSEL, AMM implemented in PyGWAS (1.7.4) |

For manuscripts utilizing custom algorithms or software that are central to the research but not yet described in published literature, software must be made available to editors and reviewers. We strongly encourage code deposition in a community repository (e.g. GitHub). See the Nature Portfolio guidelines for submitting code & software for further information.

## Data

Policy information about availability of data

All manuscripts must include a data availability statement. This statement should provide the following information, where applicable:

- Accession codes, unique identifiers, or web links for publicly available datasets
- A description of any restrictions on data availability
- For clinical datasets or third party data, please ensure that the statement adheres to our policy

Newly generated RNA-seq and bisulfite sequencing data are available at GEO under accession number GSE183785.

## Research involving human participants, their data, or biological material

Policy information about studies with human participants or human data. See also policy information about sex, gender (identity/presentation), and sexual orientation and race, ethnicity and racism.

| | |
|---|---|
| Reporting on sex and gender | no human data involved |
| Reporting on race, ethnicity, or other socially relevant groupings | no human data involved |
| Population characteristics | no human data involved |
| Recruitment | no human data involved |
| Ethics oversight | no human data involved |

Note that full information on the approval of the study protocol must also be provided in the manuscript.

# Field-specific reporting

Please select the one below that is the best fit for your research. If you are not sure, read the appropriate sections before making your selection.

☒ Life sciences ☐ Behavioural & social sciences ☐ Ecological, evolutionary & environmental sciences

For a reference copy of the document with all sections, see nature.com/documents/nr-reporting-summary-flat.pdf

# Life sciences study design

All studies must disclose on these points even when the disclosure is negative.

| | |
|---|---|
| Sample size | Published met1 and control WT RNA-seq data: 2 libraries for Col-0 inflorescence data, 6 libraries for Col-0 leaf data, 3 libraries for Col-0 seedling data, 3 libraries for each accession. |
| Data exclusions | No datasets were excluded |
| Replication | Two independent alleles of mutant plants were analysed separately where available. At least 2 biological replicates were used for RNA-seq analyses of met1. |
| Randomization | Randomization is not appropriate for this study design because samples were not allocated into experimental and control groups |
| Blinding | Blinding is not appropriate for this study design because it did not involve relevant group allocation |

# Reporting for specific materials, systems and methods

We require information from authors about some types of materials, experimental systems and methods used in many studies. Here, indicate whether each material, system or method listed is relevant to your study. If you are not sure if a list item applies to your research, read the appropriate section before selecting a response.

## Materials & experimental systems

| n/a | Involved in the study |
|---|---|
| ☒ | ☐ Antibodies |
| ☒ | ☐ Eukaryotic cell lines |
| ☒ | ☐ Palaeontology and archaeology |
| ☒ | ☐ Animals and other organisms |
| ☒ | ☐ Clinical data |
| ☒ | ☐ Dual use research of concern |
| ☐ | ☒ Plants |

## Methods

| n/a | Involved in the study |
|---|---|
| ☒ | ☐ ChIP-seq |
| ☒ | ☐ Flow cytometry |
| ☒ | ☐ MRI-based neuroimaging |

# Plants

| | |
|---|---|
| Seed stocks | Seeds were obtained from the Nottingham Arabidopsis Stock Centre (NASC) and the Arabidopsis Biological Resource Center (ABRC) as described in Methods |
| Novel plant genotypes | None |
| Authentication | None required |
